# Climate Warming Since the Holocene Accelerates West–East Communication for the Eurasian Temperate Water Strider Species *Aquarius paludum*

**DOI:** 10.1093/molbev/msac089

**Published:** 2022-04-28

**Authors:** Zhen Ye, Juanjuan Yuan, Jakob Damgaard, Gavril Marius Berchi, Fabio Cianferoni, Matthew R. Pintar, Horea Olosutean, Xiuxiu Zhu, Kun Jiang, Xin Yang, Siying Fu, Wenjun Bu

**Affiliations:** Institute of Entomology, College of Life Sciences, Nankai University, 94 Weijin Road, Tianjin 300071, China; College of Life Sciences, Zaozhuang University, 1 Beian Road, Shandong 277000, China; Natural History Museum of Denmark, Zoological Museum, Universitetsparken 15, 2100 Copenhagen Ø, Denmark; Department of Taxonomy & Ecology, Faculty of Biology & Geology, Babeş-Bolyai University, 5–7 Clinicilor Street, 400015 Cluj-Napoca, Romania; Institute for Advanced Environmental Research, West University of Timișoara, 4 Oituz Street, 300086 Timișoara, Romania; Research Institute on Terrestrial Ecosystems, National Research Council of Italy, Via Madonna del Piano 10, I-50019 Sesto Fiorentino, Italy; Zoology, “La Specola”, Natural History Museum, University of Florence, Via Romana 17, I-50125 Florence, Italy; Institute of Environment, Florida International University, Miami, FL, USA; Applied Ecology Research Center, Lucian Blaga University of Sibiu, 5-7 Ion Ratiu Street, 550012 Sibiu, Romania; Institute of Entomology, College of Life Sciences, Nankai University, 94 Weijin Road, Tianjin 300071, China; Institute of Entomology, College of Life Sciences, Nankai University, 94 Weijin Road, Tianjin 300071, China; School of Sports, Taiyuan University of Science and Technology, 66 Waliu Road, Shanxi 030024, China; Institute of Entomology, College of Life Sciences, Nankai University, 94 Weijin Road, Tianjin 300071, China; Institute of Entomology, College of Life Sciences, Nankai University, 94 Weijin Road, Tianjin 300071, China

**Keywords:** aridification, climate warming, Eurasia, transcontinental temperate species, hybrid zone, Pleistocene glaciations

## Abstract

Holocene climate warming has dramatically altered biological diversity and distributions. Recent human-induced emissions of greenhouse gases will exacerbate global warming and thus induce threats to cold-adapted taxa. However, the impacts of this major climate change on transcontinental temperate species are still poorly understood. Here, we generated extensive genomic datasets for a water strider, *Aquarius paludum*, which was sampled across its entire distribution in Eurasia and used these datasets in combination with ecological niche modeling (ENM) to elucidate the influence of the Holocene and future climate warming on its population structure and demographic history. We found that *A*. *paludum* consisted of two phylogeographic lineages that diverged in the middle Pleistocene, which resulted in a “west–east component” genetic pattern that was probably triggered by Central Asia-Mongoxin aridification and Pleistocene glaciations. The diverged western and eastern lineages had a second contact in the Holocene, which shaped a temporary hybrid zone located at the boundary of the arid–semiarid regions of China. Future predictions detected a potentially novel northern corridor to connect the western and eastern populations, indicating west–east gene flow would possibly continue to intensify under future warming climate conditions. Further integrating phylogeographic and ENM analyses of multiple Eurasian temperate taxa based on published studies reinforced our findings on the “west–east component” genetic pattern and the predicted future northern corridor for *A*. *paludum*. Our study provided a detailed paradigm from a phylogeographic perspective of how transcontinental temperate species differ from cold-adapted taxa in their response to climate warming.

## Introduction

After the last ice age ended, our planet entered a period with a stable, warm climate during the Holocene (Dansgaard et al. 1993; deMenocal et al. 2000). This warming climate has accelerated under human-induced greenhouse gas emissions since the mid-20th century, which has resulted in a worldwide threat to biodiversity (Omann et al. 2009). Many studies have shown that global warming has already increased the risk of extinction, especially for cold-adapted taxa that are sensitive to environmental changes (Lorenzen et al. 2011; Cooper et al. 2015; Fedorov et al. 2020; Louis et al. 2020). In contrast, transcontinental temperate species are expected to have a greater tolerance to warmer temperatures and therefore might be more flexible in their responses to climate warming (Gottfried et al. 2012). Temperate species with broad distributions usually have distinct intraspecific, phylogeographic lineages that are likely to have wider ecological breadths; hence, they might be able to cope with a broader range of temperature shifts and even expand their ranges under future warming climate conditions, which would potentially lead to hybridization among phylogeographic lineages (Bolnick et al. 2011; Ye, Chen, et al. 2020). An informative approach for predicting the biological consequences of ongoing global warming in the near future is to investigate the impacts of future climate scenarios on species through ecological niche modeling (ENM), in which the suitable climatic envelope of a species is modeled from species occurrence records and climate data before being projected across space and/or through time (Capblancq et al. 2020). Moreover, population genetics has been used to reveal genetic architectures, such as genetic diversities, population structures, and demographic histories, and such data have been used to evaluate the genetic responses to climate warming for many cold-adapted species (e.g., Theodoridis et al. 2018; Fedorov et al. 2020; Louis et al. 2020). However, compared with the cold-adapted taxa, there are still few case studies that have been performed to study the responses of transcontinental temperate species to climate warming by combining ENM and population genetics.

Eurasia is a supercontinent that joins the European and Asian continents and accounts for ∼36.2% of Earth’s total land area (Usoltsev et al. 2020). This supercontinent has experienced drastic climate changes since the mid-Miocene Climatic Optimum (Zachos et al. 2001), when Central Asia was transitioning to an arid environment. Thereafter, the arid conditions of Central Asia continued during the Pleistocene (Favre et al. 2015). The expanded aridification in the vast interior of the supercontinent from the mid-Miocene has gradually split this giant landmass into west–east differentiated continents. Furthermore, Pleistocene glaciation cycles began at ∼2.58 Ma, which led to cooler and drier conditions throughout the vast interior of Eurasia (Favre et al. 2015). These two major climate events significantly impacted Palaearctic biotas, which resulted in a “west–east component” genetic pattern for some Eurasian temperate organisms, such as birds (e.g., *Pica pica*, Song et al. 2018), mammals (e.g., *Meles meles*, Marmi et al. 2006), and invertebrates (e.g., *Argiope bruennichi*, Krehenwinkel et al. 2016). However, as the temperatures have begun to rise since the Holocene, it remains unclear whether transcontinental genetic exchanges could occur between the western and eastern phylogeographic lineages and whether such hybrid zones are associated with secondary contact. One of the constraints for further studies of this hypothesis is that the conclusions from recent phylogeographic studies of Eurasian temperate species have mainly relied on mitochondrial DNA. Due to the maternal inheritance of mitochondria, the genetic information contained in mitochondrial DNA cannot fully represent the evolutionary history of both parents, which might ignore the discoveries of hybrid populations and bias inferences on the true complexity of demographic histories (Andrews et al. 2016; Hinojosa et al. 2019; Ye, Yuan, et al. 2020). Recent developments in high-throughput sequencing techniques have provided the ability to identify genetic structures and hybridization at an unprecedented level, assess demographic histories with much greater robustness, and identify candidate loci under divergent natural selection in nonmodel taxa (Andrews et al., 2016). These advancements will greatly enhance our understanding of the underlying phylogeographic mechanisms that are involved in shaping the “west–east component” genetic pattern of Eurasian temperate species.

*Aquarius paludum* (Fabricius), a semiaquatic insect, has a wide distribution in the low and middle latitudes (1–50°N) of Eurasia, where it is geographically distributed from western Europe (England) to East Asia (Japan), which is typical of transcontinental temperate species (Andersen 1990). There are no significant color or size differences and no specific differences in the male genital structures among the specimens from western Europe and East Asia, which justify its consideration as a widespread Eurasian species (Andersen 1990). It prefers lentic habitats and has often been recorded in large stagnant water bodies, such as ponds, canals, slow-flowing stretches of rivers, lakes, rain pools, and paddy fields (Andersen 1990; Chen et al. 2005; Cianferoni and Mazza 2012). Most individuals are macropterous and can disperse over relatively great distances. A molecular phylogenetic analysis of the *A*. *paludum* group that involved multiple individuals of *A*. *paludum* using a 425 bp COI gene fragment suggested that *A*. *paludum* consisted of two genetic lineages that correspond to the western and eastern regions of its distribution range (Damgaard and Zettel 2003), which was consistent with the “west–east component” genetic patterns found in other Eurasian temperate organisms (e.g., Marmi et al. 2006; Wu et al. 2015; Krehenwinkel et al. 2016; Fields et al. 2018; Song et al. 2018). In addition, a physiological and behavioral study established that *A*. *paludum* could change its reproductive and dispersal characteristics in response to climate warming (Harada et al. 2013). These findings make *A*. *paludum* an ideal case study for examining whether climate warming since the Holocene is an important driver for promoting west–east communication in this transcontinental temperate species.

In the present study, we adopted comprehensive sampling and generated a variety of datasets to test this hypothesis. We used a combination of mitochondrial fragments (COI + COII), whole mitochondrial genome (13 mitochondrial protein-coding genes, PCGs), double-digest restriction site-associated DNA sequencing (ddRAD-seq), and whole-genome sequencing (WGS) to infer population structure and demographic history, whereas searching for outlier loci associated with lineage differentiation of *A*. *paludum*. We then utilized morphological diagnostic characteristics and climatic variables to examine differences in external morphology and to quantify niche comparisons between the divergent lineages. Finally, we conducted phylogeographic and ENM analyses of multiple Eurasian temperate taxa based on the novel dataset and published studies to reveal phylogeographic patterns and simulated their responses to future climate warming under minimum and maximum greenhouse gas emission scenarios.

## Results

### Mitochondrial DNA Sequencing

A total of 1,280 bp of the mitochondrial PCGs were obtained from 390 individuals, including segments of the COI (647 bp) and COII (633 bp) genes. The mitogenome contained 13 typical mitochondrial PCGs, and the lengths of the mitochondrial PCGs ranged from 156 bp (ATP8) to 1,713 bp (ND5). The concatenated matrix of the 13 mitochondrial PCGs included 10,960 aligned nucleotide sites representing 112 individuals from 22 populations.

### Nuclear SNP Calling

For the ddRAD-seq dataset, 249 individuals were sequenced, which resulted in 1,375,147,138 raw reads. The number of reads that were mapped to each individual ranged from 1,494,153 to 22,973,092, with an average of 5,522,679 reads per individual. A total of 1,364,149,347 clean reads were retained after the low-quality reads were filtered out. The ddRAD_95 SNPs dataset contained 1,179 ddRAD-seq loci, 32,114 SNPs and 2.98% missing sites, from which 1,179 unlinked SNPs were randomly selected and constituted the ddRAD_95 USNPs dataset (generated by ipyrad software). For the whole-genome sequencing dataset, the mapping of 21 resequenced data points to the reference genome of *Gerris buenoi* resulted in an average depth and breadth of coverage of 7.16 and 99.4%, respectively, with an average mapping rate of 44.2%. We finally identified a total of 510,262 high-quality SNPs after filtering.

### Genetic Polymorphism, Population Genetic Structure, and Mantel Test

**Fig. 1.**
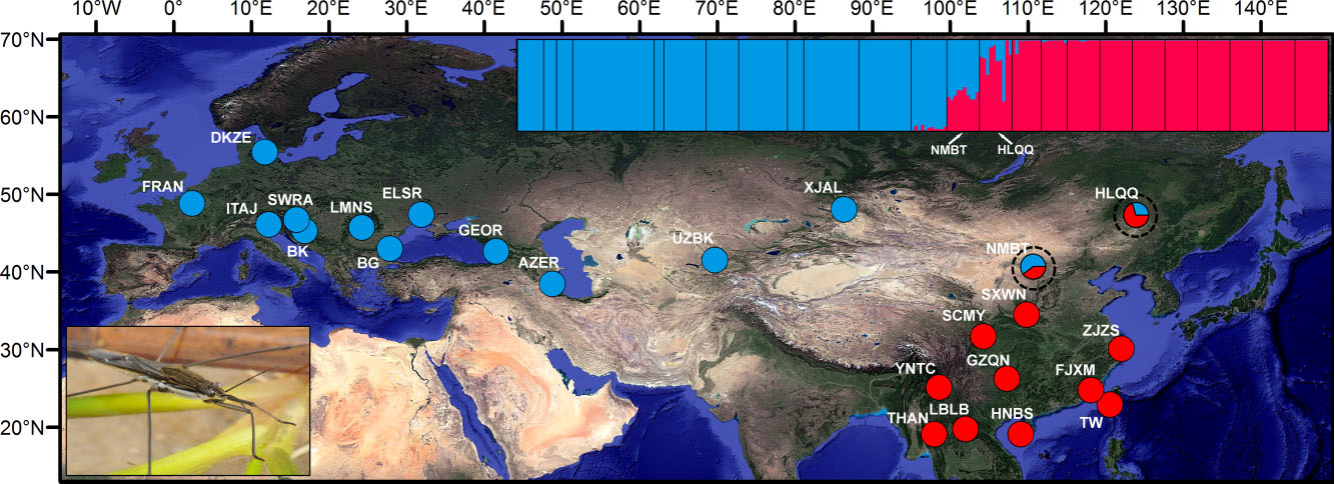
Sampling of *A*. *paludum* throughout Eurasia for ddRAD-seq analyses and population structure estimated by the STRUCTURE method for *K* = 2 based on the ddRAD_95 USNPs dataset (upper right). The sample locations are colored according to the genetic lineages, which are color-coded by the population structure. Black dotted circles indicate the detected hybrid populations (NMBT and HLQQ). The photographic image of *A*. *paludum* is from a copyrighted website (http://www.biopix.com/).

The measures of genetic polymorphisms including *H_O_*, *H_E_*, and *π_S_* estimated based on the 32,114 SNPs of the ddRAD_95 SNPs dataset are shown in supplementary table S5, Supplementary Material online. The *π_S_* value was higher in the eastern lineage, whereas the *H_O_* and *H_E_* values of the western lineage exhibited relatively high heterozygosities. Our BayeScan analysis identified 74 outlier SNPs that had significantly elevated *F*_ST_ values (supplementary fig. S2, Supplementary Material online), and we removed them and therefore retained the 1,105 neutral SNPs for the downstream analyses. In the STRUCTURE analysis, the plot of ΔK against a range of *K* values (from 1 to 10) revealed a distinct peak at *K* = 2 (fig. 1). Two genetic lineages, a western lineage (i.e., AZER, BGLY, BKKL, DKZE, ELSR, FRAN, GEOR, ITAJ, SWRA, LMNS, UZBK, XJAL, and NMBT) and an eastern lineage (i.e., HLQQ, SXWN, SCMY, HNBS, LBLB, THAN, TWYT, GZQN, FJXM, YNTC, and ZJZS), were revealed. The NMBT and HLQQ populations exhibited heterozygosity that incorporated the western and eastern lineages (fig. 1). The combination of admixture clustering using snmf and discriminant analysis of principal components (DAPC) also allowed us to identify this fine-scale genetic structure based on the nuclear SNPs data (supplementary figs. S3 and S4, Supplementary Material online). Within their respective lineages, STRUCTURE analysis determined that *K* = 3 and *K* = 2 were the best-fitting numbers for the western and eastern populations, respectively (supplementary fig. S5, Supplementary Material online). For the mitochondrial data, Bayesian analysis of population structure (BAPS) analysis identified the two genetic lineages, which was in accordance with the results based on the nuclear SNPs dataset (supplementary fig. S6, Supplementary Material online). However, the hybrid populations (i.e., NMBT and HLQQ) detected by the nuclear SNPs dataset were not supported by the BAPS analysis based on the mitochondrial data, which were both grouped into the eastern lineage (supplementary fig. S6, Supplementary Material online). The hierarchical analysis of molecular variance (AMOVA) results identified significant genetic differentiation when the populations were divided into two lineages (i.e., western and eastern lineages) (supplementary table S6, Supplementary Material online).

The Mantel tests indicated significant correlations between the genetic and geographic distances (*r* = 0.6483, *P* = 0.001), genetic and environmental distances (*r* = 0.4226, *P* = 0.001), and genetic and cost distances (*r* = 0.5323, *P* = 0.001) (supplementary fig. S7, Supplementary Material online). The least cost distances (LCDs) analysis suggested that the least-cost paths for *A*. *paludum* occurred mostly in Europe and East Asia for the current and future conditions (supplementary fig. S8, Supplementary Material online). A novel northern dispersal corridor between the western and eastern populations was identified under the maximum greenhouse gas emission scenario for the year 2070 (supplementary fig. S8, Supplementary Material online).

### Demographic Model Testing

The demographic model selection strongly indicated that model M6 was the best fit to the observed data with Akaike information criterion (AIC) weight = 1 (supplementary table S7, Supplementary Material online), which favored an initial divergence between the western and eastern lineages with no gene flow, followed by a recent secondary contact with bidirectional gene flows between the two lineages. All other models were poorly supported (AIC weights = 3.75147e−84–7.879677e−21, supplementary table S7, Supplementary Material online).

**Fig. 2.**
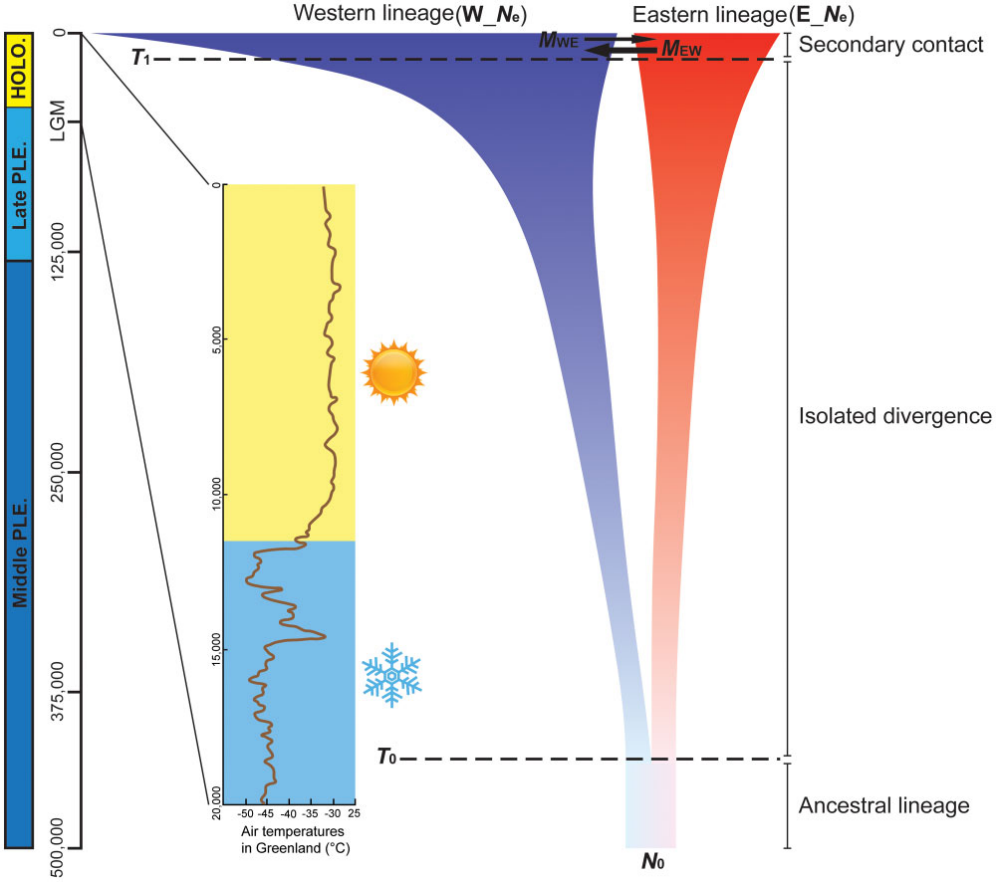
Reconstruction of the best-fit demographic model for *A*. *paludum* (Model 6). The vertical chronological coordinates are referenced against the International Chronostratigraphic Chart (Cohen et al. 2013). The magnified graph from the LGM to date indicates the reconstructed air temperatures from the GISP 2 Ice core in Greenland (brown solid line, Platt et al. 2017). Images of sun and snowflake showed that the climate was warm during the Holocene and cold during the late Pleistocene. *N*_0_, W_*N*_e_, and E_*N*_e_, effective population size estimates of the ancestral, western, and eastern lineages, respectively; *T*_0_, divergence time; *T*_1_, contact time; black arrows represent relative estimates of the migration probabilities, with variation in the arrow size with magnitude, and the estimated migration rates (*M*_EW_, *M*_WE_) are labeled near the arrow.

Point estimates of the demographic parameters for model M6 were provided with 95% confidence intervals (CIs) (fig. 2, supplementary table S8, Supplementary Material online). The *N*_e_ values of the western lineage (W_*N*_e_ = 7,024,866, 95% CIs: 6,285,572–7,764,159) were much higher than those of the eastern lineage (E_*N*_e_ = 549,728, 95% CIs: 491,442–608,014), both of which were much higher than the estimates for the ancestor of the two lineages (*N*_0_ = 5,666, 95% CIs: 4,940–6,393). The estimated mean times indicated that the western lineage first split from the eastern lineage 383,708 ya (95% CIs: 343,278–424,139 ya), and the two diverged lineages had a second contact of 2,870 ya (95% CIs: 2,529–3,212 ya). Estimates of the migration probabilities per generation between the two lineages were asymmetric, with high probabilities observed for migration of the eastern lineage into the western lineage at 1.2E−6 (1.1E−6–1.3E−6) and low probabilities for migration of the western lineage into the eastern lineage at 3.6E−7 (3.4E−7–3.8E−7).

### Historical Demographic Changes

**Fig. 3.**
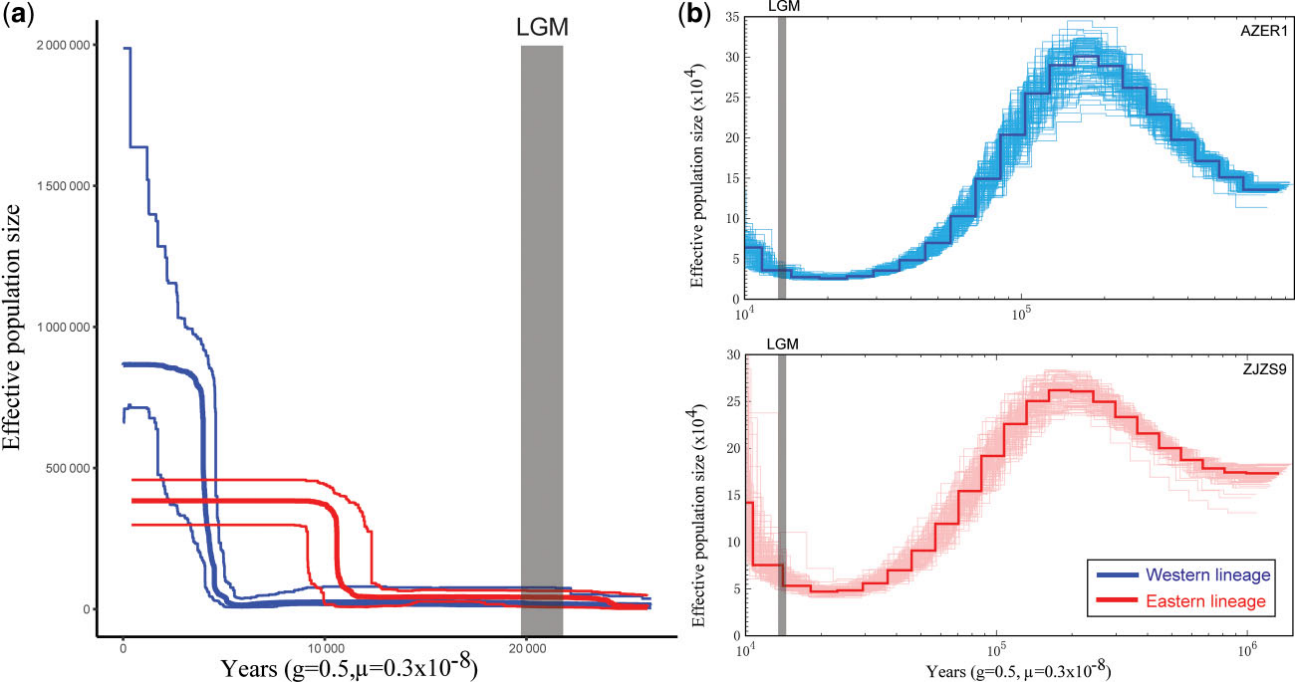
(*a*) Historical demographic changes of the two lineages based on the nuclear SNPs data inferred from stairway plots. (*b* and *c*) Historical demographic changes estimated in the PSMC model of two individuals (AZER1 and ZJZS9) representing two genetic lineages (western lineage = thick blue lines, eastern lineage = thick red lines) for *A*. *paludum.* Thin lines represent the 95% CIs. The *X*-axis is the time scale before the present. The *Y*-axis is the estimated effective population size. The gray shaded lines indicate the LGM period.

The stairway plot analysis showed that the western and eastern lineages expanded rapidly 5,000 and 10,000 ya after the last glacial maximum (LGM), respectively, and eventually maintained stable effective population sizes until now (fig. 3*a*). The pairwise sequential Markovian coalescent (PSMC) analysis indicated that the demographic history of *A*. *paludum* could be traced back to ∼1 Ma, and the western and eastern lineages both experienced similar demographic histories, which exhibited recent population declines that were most likely caused by the LGM, which supports the results of the stairway plot (fig. 3*b*). However, when the analysis of the demographic dynamics accounted for the mitochondrial data, the BSP plots could not reject population stability since the LGM for the two genetic lineages (supplementary fig. S9, Supplementary Material online).

### Niche Comparison

**Fig. 4.**
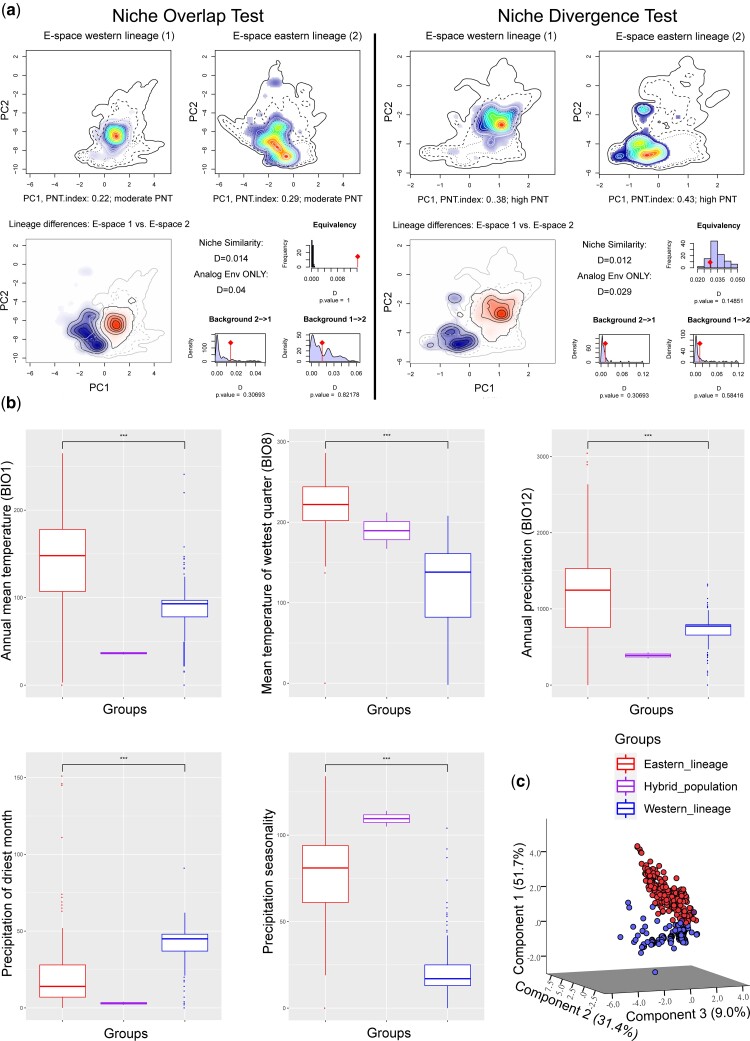
(*a*) Humboldt results including the NOT and NDT; (*b*) boxplots of the five most important climatic variables (BIO1, BIO8, BIO12, BIO14, and BIO15) comparing the western and eastern lineages together with their hybrid populations of *A*. *paludum*. Values significant at *P* < 0.001 are marked with asterisks; (*c*) PCA of five climatic variables associated with the occurrence of the two lineages and their hybrid populations of *A*. *paludum*.

The results of Humboldt indicated that both the equivalency test and background test were nonsignificant, meaning that the occupied environmental spaces of the western and eastern lineages were not significantly different (fig. 4*a*). However, the niche overlap test (NOT) and niche divergence test (NDT) might be inconclusive due to the relatively high potential niche truncation index of our analyses (PNTI), which suggested that the measured occupied niche might not reflect the species’ fundamental niche due to niche truncation driven by the limited available E-space and might have led to the inability to obtain appropriate background data. Therefore, we further explored the five most informative climatic variables identified by Humboldt. We found that the western lineage of *A*. *paludum* tended to occupy habitats with lower temperatures and less precipitation than the eastern lineage (fig. 4*b*). The hybrid populations (i.e., NMBT and HLQQ) fell into the intermediate environmental space with respect to the mean temperature of wettest quarter (fig. 4*b*). A principle component analysis (PCA) of the pooled environmental variables revealed reduced significant components, thus defining a realized niche space occupied by the two lineages and hybrid populations (fig. 4*c*). The first three components of the PCA together explained 92.2% of the overall variance. The first component (PC-1) was closely associated with the mean temperature of the wettest quarter and precipitation seasonality, whereas the second component (PC-2) was associated with the precipitation of the driest month (supplementary table S9, Supplementary Material online). The climate space occupied by the western lineage departed from that occupied by the eastern lineage mainly with respect to component 1 (supplementary table S9, Supplementary Material online).

### Scans for Selection Signatures

In our pcadapt analysis, we detected 5,610 outlier SNPs out of 510,262 SNPs (1%) by using a false discovery rate (FDR) of 0.05 based on the three cut-off methods (supplementary fig. S10*a* and *b*, Supplementary Material online). The gene ontology (GO) enrichment analysis identified 57 significantly enriched GO terms (FDR < 0.05) corresponding to 1,293 outlier sequences. These sequences covered a wide range of functions in biological processes: metabolic process, cellular process, regulation of biological process, biological regulation, response to stimulus, signaling, localization, cellular component organization, or biogenesis (supplementary fig. S1*c*, Supplementary Material online).

### Morphological Cluster

In the PCA of eight morphological characteristics, a visual inspection revealed that the two genetic lineages could not segregate with respect to components 1 and 2 (supplementary fig. S11, Supplementary Material online). The compared boxplots indicated that the western lineages of *A*. *paludum* males tended to have wider bodies and slightly shorter seventh abdominal sterna and connexival spines (supplementary fig. S12, Supplementary Material online).

### Phylogeographic and Ecological Niche Analysis of Multiple Eurasian Temperate Species

**Fig. 5.**
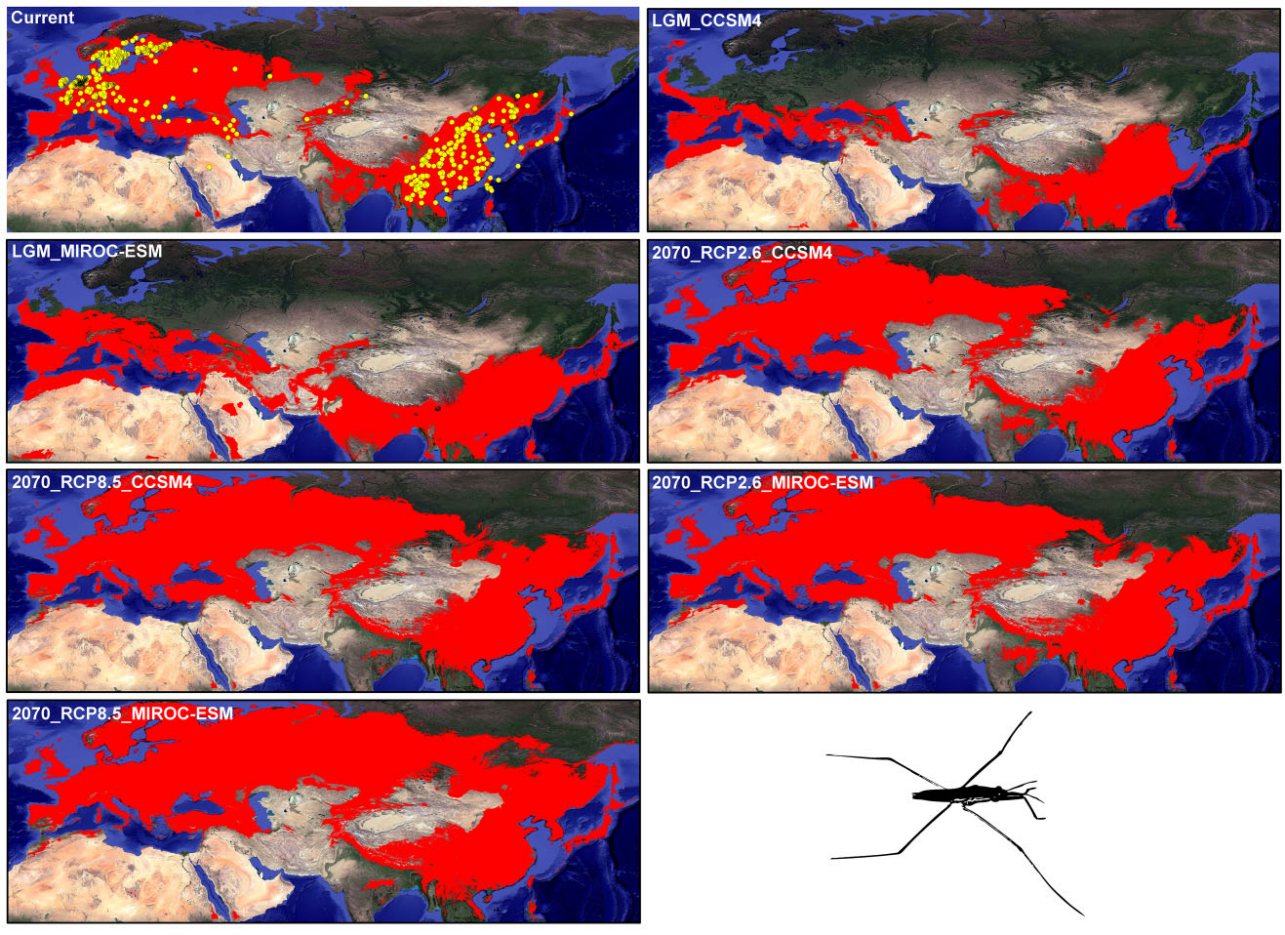
Modeled suitable areas of *A. paludum* throughout the Eurasia under the 10 percentile training presence threshold from the current climatic condition, the LGM, and the two greenhouse gas emission scenarios (RCP 2.6 and RCP 8.5) for the year 2070 under the CCSM4 and MIROC-ESM. Red colors indicate habitat suitability areas. Occurrence localities (yellow dots) are used for ecological niche modeling.

The Eurasian temperate species all exhibited deep west–east differentiation patterns, and the divergence times between the western and eastern populations occurred in the Pleistocene (i.e., 0.29–2.27 Ma) (supplementary fig. S13, Supplementary Material online). For the ENM analyses, the best-fit combinations of the feature classes and regularization multipliers of the six Eurasian temperate species were used (supplementary fig. S14, Supplementary Material online). The niche model exhibited good predictive performance (mean AUC = 0.81–0.92). For *A*. *paludum*, highly suitable areas were observed in most of Europe, the Central Asian piedmont across northwestern Xinjiang, the southern foot of the Himalayas, northern India, and East Asia (fig. 5). The unsuitable areas were mainly located in the Central Asia-Mongoxin arid region (“Mongoxin” specifically refers to the region covering Inner Mongolia, Alxa, Tarim, Qaidam, Junggar basin, and Tianshan Mountains) and Qinghai-Tibet Plateau (fig. 5). Integrating the current niche model into the simulated LGM climate conditions suggested that the potential range of *A*. *paludum* contracted greatly toward the south in Europe and East Asia (below 50°N) (fig. 5). The future predictions suggested that the highly suitable habitats expanded moderately to northern Eurasia, thus forming a potentially novel northern corridor to connect the western and eastern populations, especially under the maximum greenhouse gas emission scenario representative concentration pathways (RCP) 8.5 (fig. 5). Under the past and future climate scenarios, the environmental anomaly area (<0, black area) in the entire potential distribution region was small (supplementary fig. S15, Supplementary Material online). The average similarity values of the 212 record points for *A*. *paludum* were >0 under different predicted climate scenarios. The current suitable areas for the other five Eurasian temperate species were similar to that of *A*. *paludum*, indicating that the highly suitable habitats were mainly distributed in most of Europe and East Asia, together with unsuitable areas being present in the Central Asia-Mongoxin arid region and Qinghai-Tibet Plateau (supplementary figs. S16–S21, Supplementary Material online). After projecting the current niche into the LGM historical climate condition, all six Eurasian temperate taxa exhibited consistently contracted patterns (below 50°N) (supplementary fig. S16, Supplementary Material online). Under future climate scenarios, three of the five Eurasian temperate species, namely, *Daphnia magna*, *Lymantria dispar*, and *P. pica*, exhibited similar or expanded corridors as suitable areas between the western and eastern regions in northern Eurasia (supplementary fig. S16, Supplementary Material online).

## Discussion

### Central Asia-Mongoxin Aridification and Pleistocene Glaciations Triggering a “West–East Component” Genetic Pattern

Our population structure analyses based on a variety of types of molecular data indicated that *A. paludum* consisted of two phylogeographic lineages (i.e., western and eastern lineages), and AMOVA analyses identified strong genetic differentiation when the populations were divided into the two lineages. In addition, our selected demographic model strongly favored an initial divergence between the western and eastern lineages with no gene flow, and the divergence between the western and eastern lineages occurred at 383,708 ya (95% CIs: 343,278–424,139 ya) in the middle Pleistocene. These results reinforced the “west–east component” genetic pattern of *A*. *paludum*, which was consistent with the conclusion from previous phylogenetic studies using a 425 bp COI gene fragment (Damgaard and Zettel 2003). We suggest that this “west–east component” genetic pattern was most likely caused by habitat fragmentation because of the aridification and climatic cooling during the middle Pleistocene (i.e., 0.12–0.78 Ma, Cohen et al. 2013). The aridification in the vast interior of Eurasia was mainly caused by the rapid uplift of the Qinghai-Tibet Plateau and the regression of the Paratethys Sea. In the Late Eocene (i.e., 34–37 Ma), the collision between the Indian and Asian Plates caused the uplift of the Tibetan Plateau, which disrupted the water exchange between Central Asia and East Asia (Rögl 1998; Hou et al. 2011). Afterwards, as the African and Indian Plates moved northwards and converged with the Eurasian Plate, which resulted in the rapid regression of the Paratethys Sea, aridification appeared in Central Asia and persisted throughout the Pleistocene (Rögl 1998; Hou et al. 2011). Meanwhile, a dramatic climate shift, termed the “mid-Pleistocene revolution” (i.e., 0.9 Ma), occurred in the Pleistocene epoch (Willeit et al. 2019). Around this time, climate oscillations shifted from the 41,000-year cycles of the earlier Pleistocene to the 100,000-year cycles of the later Pleistocene (Huang et al. 2005). The later cycles were characterized by the increasing severity and duration of cold and dried climates, thus intensifying the aridification of Central Asia, and they were thought to have had profound impacts on the biota, especially in Eurasia (Head and Gibbard 2005). For instance, a major faunal turnover was detected in Europe and East Asia around the time of the climate transition (Wang et al. 2000; Palombo et al. 2005).

This “west–east component” genetic pattern was further supported by our phylogeographic analyses using multiple Eurasian temperate species. All six Eurasian temperate species exhibited consistent and deep west–east differentiation patterns, and they were likely isolated by the broad Central Asia-Mongoxin arid region. The estimated divergences between the western and eastern populations all occurred in the Pleistocene (i.e., 0.29–2.27 Ma), and it is likely that these species would have been affected by the Pleistocene glaciations and forced to migrate into the southern warmer refuges, which further limited gene flow between the western and eastern populations and deepened the “west–east component” genetic pattern. Although we could not test this hypothesis by using ENM analysis due to a lack of paleoclimatic data for the early and middle Pleistocene, the potentially suitable areas we simulated under the LGM in the late Pleistocene might provide analogous evidence, thus indicating that the suitable areas for all six Eurasian temperate species contracted greatly toward the southern refuges (i.e., below 50°N) in western Europe and East Asia during the LGM period. Combined with the above analyses, we suggest that the broad, arid habitats of Central Asia-Mongoxin acted as an isolating barrier together with the cold, dry climate in the Pleistocene inducing western and eastern population divisions for the Eurasian temperate species, which finally shaped a “west–east component” genetic pattern.

### Climate Warming Since the Holocene Shaped a Novel Northern Corridor to Promote West–East Gene Flow

The level of climate warming is predicted to dramatically alter the spatial distribution of global biodiversity (McGaughran et al. 2021). For example, cold-adapted Arctic taxa would move toward the poles or to higher altitudes to track suitable climates under climate warming (e.g., Theodoridis et al. 2018; Fedorov et al. 2020; Louis et al. 2020). Compared with the preceding glacial period, the climate was relatively warm and stable during the Holocene, especially after the Holocene climate optimum, which was a warmer period (i.e., 5,000–9,000 ya) that has been suggested to have experienced temperature increases of up to 4 °C in the northern regions of Eurasia (Koshkarova and Koshkarov 2004). Our optimal demographic model suggested that the diverged western and eastern lineages of *A. paludum* experienced a recent secondary contact at 2,870 ya (95% CIs: 2,529–3,212 ya). Further STRUCTURE and DAPC analyses detected two populations (i.e., NMBT and HLQQ) that showed hybrid types incorporating the western and eastern lineages, and they provided direct genetic evidence for the contact between the western and eastern populations under the Holocene climate warming conditions. The detected second contact area belongs to a transition zone that lies at the boundary of the arid–semiarid regions of China near the “Heihe-Tengchong Line,” which is characterized by a variety of complex environmental landscapes (Wang et al. 2020). This climatic boundary prevented further migrations between the western and eastern populations, where individuals of the western and eastern populations shaped the hybrid zone through gene exchange. In addition, hybridization between divergent lineages is regarded as an important adaptive mechanism for adjusting to complex environments (Maguilla et al. 2017). This process can provide abundant raw material for instantaneously increasing genetic variation by the transfer of adaptive alleles via gene exchange (Nelson et al. 2017). Therefore, the detected hybrid populations, NMBT and HLQQ, were expected to be more efficient in adapting to the complex environment of the transition zone than either the western or eastern lineage.

Since the beginning of the Anthropocene, humans have both directly and indirectly driven new climatic trajectories, which have resulted in hotter climatic conditions (Steffen et al. 2018). The largest driver of warming is the emissions of gases that create a greenhouse effect, and carbon dioxide (CO_2_) and methane account for more than 90% of these gases (Heede 2014). Fossil fuel burning (e.g., coal, oil, and natural gas) for energy consumption is the main source of these emissions, with additional contributions from agriculture, deforestation, and chemical reactions in certain manufacturing processes. Our LCDs analysis and future predictions suggested that the highly suitable habitats for *A*. *paludum* expanded moderately to northern Eurasia, thus forming a potential northern corridor across the broad Central Asia-Mongoxin arid region to connect the western and eastern populations, especially under the maximum greenhouse gas emission scenario (RCP 8.5). Furthermore, three of the five Eurasian temperate species, *D*. *magna*, *L*. *dispar*, and *P*. *pica*, also exhibited similar or expanded corridors as suitable areas between western and eastern Eurasia under future climatic scenarios. Coupled with the results from the ENM and population genetic analyses, we found that the west–east communications for the Eurasian temperate species would possibly continue to intensify under future warming climate conditions, and the hybrid zone due to the second contact might continue to expand or even homogenize the present “west–east component” genetic pattern.

### Mito-Nuclear Discordance Pattern in the Hybrid Populations of *A*. *paludum*

Mito-nuclear discordance has been increasingly found in phylogeographic studies within and among conspecific populations and closely related species (Avise et al. 2016). This intriguing phenomenon may be caused by various biological processes, such as female-linked selection (e.g., Fossøy et al. 2016), sex-biased dispersal (e.g., Roycroft et al. 2019), mitochondrial capture through introgression (e.g., Andersen et al. 2021), and invasions by reproductive parasites (e.g., *Wolbachia* spp., Arif et al. 2021). In this study, mito-nuclear discordance was mainly observed in the populations located in the hybrid zone, which suggested that the hybrid populations of NMBT and HLQQ incorporating both the western and eastern genetic components detected by the nuclear data were completely grouped into the eastern lineage based on the mitochondrial data. This most likely represented a special case of “male-biased dispersal,” in which males of both the western and eastern populations could mate freely, whereas females remained restricted in their movements. In other words, females from the eastern region could migrate more easily to colonize the hybrid zone and produce offspring by reproduction than females from the western region. Two pieces of evidence likely support our hypothesis. First, the hybrid zone lies at the boundary of the arid–semiarid regions of China, which are far from the western population. Our historical demographic results showed that the eastern population began to expand before the western population. These two points indicated that the eastern population might reach the hybrid zone first. Second, studies have indicated that semiaquatic bugs exhibit distinct polymorphisms in their flight apparatus, and macropterous females are usually less fertile than apterous females within a species (Andersen 1982). Therefore, we would favor an explanation for the mito-nuclear discordance in the hybrid populations of *A*. *paludum* based on the current data: in the face of rampant male dispersal, the macropterous females from the western population who later arrived at the hybrid zone relative to females from the eastern population might suffer from reduced reproductive success due to the low fertility in the established hybrid populations. However, this explanation should be further tested based on more biological evidence, such as the dispersal abilities of males and females and their reproductive behaviors.

### Contrasting Demographic History of Transcontinental Temperate Taxa vs. Cold-Adapted Taxa in Response to Climate Warming

Most transcontinental cold-adapted species are adapted to cold climates at high latitudes in northern Eurasia near the Arctic, and the magnitude of recent climate warming in high latitude regions in northern Eurasia is larger than that in temperate Eurasian regions. The influence of past or future warming events on the demographic and genetic effects of extinct and extant cold-adapted Eurasian species has been extensively studied by using paleoclimate niche modeling and phylogeographic reconstruction. The ENM predictions indicated that the decreases in suitable habitat during the Holocene climate warming contributed to the demographic decline of cold-adapted Eurasian terrestrial mammals, which led to extirpation and in some cases the extinction of taxa, such as *Coelodonta antiquitatis*, *Mammuthus primigenius*, and *Megaloceros giganteus* (Lorenzen et al. 2011; Cooper et al. 2015). For the extant cold-adapted Eurasian species, their suitable areas might further contract to the Arctic under future climate warming, as has been shown in some studies of bird and marine mammal species (e.g., Jakubas et al. 2017; Louis et al. 2020). Furthermore, most lineages within the cold-adapted Eurasian species were genetically affected by Holocene climate warming, which resulted in declines in genetic diversity and effective population sizes (Fedorov et al. 2020; Louis et al. 2020). In contrast, the transcontinental temperate species respond to climate warming quite differently. *A. paludum* is typical of transcontinental temperate species and has a wide distribution in the low and middle latitudes of Eurasia. The influence of the Central Asia-Mongoxin aridification and Pleistocene glaciations on *A. paludum* led to a “west–east component” genetic pattern. However, both the western and eastern lineages of the species have broad distribution ranges, which are likely to have wider ecological breadths and are able to cope with a broader range of temperature shifts. Therefore, the two lineages of the species are expected to adapt well to future warming climate conditions. The ENM prediction of *A. paludum* together with the other five Eurasian species confirmed this hypothesis and showed that the suitable areas for the western and eastern lineages did not contract but largely expanded under future warming climate conditions. Moreover, the demographic reconstruction of the western and eastern populations showed that the effective population sizes of the two lineages reached their maximum values during the warm interstadials in the last glacial period (∼130,000 ya), which indicated that climate warming could promote population growth. Further local adaptation studies on transcontinental temperate taxa and cold-adapted taxa are needed to understand the functions of candidate genes that adapt to temperate and cold climate conditions under the impacts of climate warming.

## Materials and Methods

### Sample Collection and DNA Extraction

We sampled 52 populations, comprising a total of 409 individuals between 1999 and 2018 across the natural distributional range of *A*. *paludum* in Eurasia. The latitude and longitude of each collection site were recorded using a handheld global positioning system (GPS) unit. All samples were preserved in 70–95% ethanol and stored in a freezer at −20 °C in the College of Life Sciences at Nankai University (NKU, Tianjin, China). Genomic DNA of each specimen was extracted from the entire body, excluding the abdomen and genitalia, of each specimen using a Universal Genomic DNA Kit (CWBIO). Detailed information for molecular voucher specimens is provided in supplementary tables S1–S4, Supplementary Material online, which includes sample codes, sample sizes, collection locations and data, longitude, and latitude.

### Mitochondrial DNA Sequencing

A total of 390 out of 409 individuals were sequenced for COI and COII fragments (supplementary table S1, Supplementary Material online). Furthermore, 112 individuals representing 22 populations were selected to generate the whole mitochondrial genome (supplementary table S2, Supplementary Material online). Details of mitochondrial DNA sequencing can be found in supplementary methods, Supplementary Material online.

### ddRAD-seq Library Preparation, Sequencing, and Variant Calling

A ddRAD-seq library was prepared to obtain nuclear SNPs following Peterson’s protocol (Peterson et al. 2012). A total of 249 individuals representing 24 populations were selected for ddRAD-seq analysis, which involved a wide geographic range and all major lineages identified by the mitochondrial data (supplementary table S3, Supplementary Material online). A ddRAD_95 SNPs dataset was generated and each locus was required to be present in at least 95% of individuals (i.e., 5% missing samples per locus). To avoid linkages across sites within the same locus, one random SNP was sampled from each locus, and the ddRAD_95 USNPs dataset was finally generated for the downstream analyses. Details of ddRAD-seq and processing of ddRAD-seq data can be found in supplementary methods, Supplementary Material online.

### Whole-Genome Sequencing and Variant Calling

To further investigate the historical demographic changes and scanning signatures of selection between the western and eastern lineages of *A*. *paludum*, we chose one individual from each population and shotgun sequenced the genomes of 21 individuals (6.51 ± 0.89 Gb, range 5.62–7.4 Gb) (supplementary table S4, Supplementary Material online). Details of whole-genome sequencing and variant calling can be found in supplementary methods, Supplementary Material online.

### Genetic Polymorphism, Population Genetic Structure, and Mantel Test

The genetic diversity based on the 32,114 SNPs of the ddRAD_95 SNPs dataset was measured by the observed heterozygosity (*H_O_*), expected heterozygosity (*H_E_*), and nucleotide diversity (*π_S_*), which were calculated in Arlequin 3.5 (Excoffier and Lischer 2010). For the nuclear SNPs dataset, we first detected outlier loci before clustering to ensure the neutrality of the markers in the 1,179 unlinked SNPs of the ddRAD_95 USNPs dataset using Bayescan 2.1 (Foll and Gaggiotti 2008). The analysis was carried out under the default parameter settings, and outlier loci were identified as those that exceeded a FDR of 0.05. The population genetic structure was inferred in the program STRUCTURE version 2.3.4 (Pritchard et al. 2000) and the R package landscape and ecological association (LEA) (Frichot and François 2015). In the STRUCTURE analysis, we used the admixture model without prior information on population membership, and the number of clusters (*K*) was set from 1 to 10. For each value of *K*, we performed five independent runs with 1,200,000 iterations following a burn-in of 200,000 replications. The optimal *K* was then determined using the delta *K* method of Evanno et al. (2005) as implemented in STRUCTURE HARVESTER (Earl and vonHoldt 2012). We further performed the STRUCTURE analysis within the respective lineages (i.e., the western and eastern lineages, see Results) using the same parameter settings. In the LEA analysis, we calculated the ancestry coefficients for 1–10 ancestral populations (*K*) using 50 replicates for the runs for each value of *K* and an alpha value of 250. In addition, the population structure based on the nuclear SNPs data was estimated by DAPC, which is a method that has no assumptions for evolutionary models, using the R package Adegenet (Jombart 2008). For the mitochondrial data, Bayesian analysis of the population structure model for clustering of individuals was implemented in BAPS 6.0 (Cheng et al. 2013), with *K* varying from 1 to 10. The best partition of the populations into *K* clusters with the highest marginal log-likelihood after ten replicates was chosen as the most representative. Finally, the hierarchical AMOVA was estimated with our best assessment of the hierarchical population structure (*K* = 2, see Results) based on 5,000 permutations to quantify how genetic variation was partitioned across the different levels of sampling using Arlequin 3.5 (Excoffier and Lischer 2010).

Isolation by distance, isolation by environment, and isolation by resistance-climate tests were investigated based on the ddRAD_95 SNPs dataset. Details of these tests can be found in supplementary methods, Supplementary Material online.

### Demographic Model Testing

Based on the results of the population genetic structure estimated from the nuclear SNPs dataset, 24 populations were grouped into the two lineages (W = western lineage, E = eastern lineage) corresponding to geography (western and eastern regions). We simulated the demographic history of *A*. *paludum* in a temporal framework and built 10 demographic models, which were all variants of the two population isolation–migration models (supplementary fig. S1, Supplementary Material online). Models included isolation only (M1), symmetric, or unidirectional migration between the two lineages (M2–M4), and symmetric or unidirectional migration between the two lineages either historically or recently (M5–M10). We selected and parameterized the best-fit demographic model using fastsimcoal2 v2.6 (Excoffier et al. 2013). This method uses coalescent simulations to approximate an expected site frequency spectrum (SFS) and a composite likelihood approach for parameter optimizations. We used a nuclear mutation rate of 3.5E−9 per site per generation following the estimates for *Drosophila melanogaster* (Keightley et al. 2009) and assuming a generation time of 0.5 years (Andersen 1982). We used easySFS (https://github.com/isaacovercast/easySFS) to generate the observed SFS and chose to project the full unlinked SNPs dataset to reduce the possibility of linkage. We performed 50 replicate runs for each model with each replicate using 50,000 coalescent simulations and ran 40 optimization cycles. The monomorphic sites were ignored to avoid overfitting. To select the best-fit model, MaxObsLhood was compared with MaxEstLhood for the data in each model for each replicate run. The best-fit run was identified as the one that had the smallest difference between MaxObsLhood and MaxEstLhood for each of the 50 replicate runs for each model. To generate 95% CIs of the parameter estimates for our best-fit model, a combination of the initial model selection runs and parametric bootstrapping were used. We simulated 100 replicates SFS from the *_maxL.par file for the best-fit run. We then performed 50 replicate analyses as described above for each of the 100 newly simulated SFS files. Finally, we calculated the mean parameter estimates and 95% CIs from the 100 best-fit bootstrapping replicates.

### Historical Demographic Changes

We used three datasets to reconstruct the recent demographic trajectories for the western and eastern lineages of *A*. *paludum*. Details of historical demographic reconstruction can be found in supplementary methods, Supplementary Material online.

### Niche Comparison

To quantify the niche comparisons between western and eastern lineages identified by the genetic structure (see Results), the same clipped layers of five climatic variables (BIO1, BIO8, BIO12, BIO14, and BIO15) and 1,643 occurrence records (1,382 for western lineage and 261 for eastern lineage) were used. The NOT and NDT were performed using corrected e-space across the full distribution and shared e-space in Humboldt (Brown and Carnaval 2019). Furthermore, we looked for differences in the five climatic variables for the western, eastern, and their hybrid occurrence records and assigned each lineage by extracting environmental data for each point and comparing them with boxplots using ggplot2 (Wickham 2009). Two record points (NMBT and HLQQ) in the potential hybridization zone were integrated in this analysis. Additionally, direct PCA was used to visualize the relationships among the two lineages together with their hybrid populations and statistically tested using the independent-samples test method in SPSS (IBM 2009).

### Scans for Selection Signatures

To test for signatures of selection between western and eastern lineages of *A*. *paludum*, we searched for outlier SNPs using R package pcadapt 4.0 (Privé et al. 2020). Outlier loci were blasted to the reference genome of *Gerris buenoi* (GenBank accession no.: GCA_001010745.2) to identify nearby genes and identified enriched GO terms. This analysis was performed using the WGS_SNPs dataset including 21 individuals (13 from the western lineage and 8 from the eastern lineage). We chosed a cut-off for outlier detection based on three methods (i.e., *q*-values, Benjamini–Hochberg procedure, and Bonferroni correction) from the less to the more conservative one. The SNPs that were identified as significant were located at the positions of the reference genome. To estimate the biological functions of the genes linked to outlier SNPs, gene sequences were extracted within 2000 bp, including the outlier SNPs, and then used for homologous search analysis. Loci under putative selection were functionally annotated by Blast2GO (Conesa et al. 2005) against all available nucleotide databases with an *E*-value cut-off of 10^−6^. Enrichment of GO terms was conducted using the program web gene ontology annotation plot (WEGO) (Ye et al. 2006) with default parameters.

### Morphological Cluster

A total of 233 male specimens representing western (83 individuals) and eastern lineages (150 individuals) were analyzed based on size measurements of morphological diagnostic characteristics. We did not include individuals of potential hybrid populations (NMBT and HLQQ) in this analysis. Eight diagnostic characteristics were measured: body length, body width, head length, pronotum length, hind femur length, abdomen length, seventh abdominal sternum length, and connexival spine length. All measurements were obtained with a stereomicroscope and recorded in millimeters. PCA based on the eight morphological variables was used to visualize the morphological relationships between the western and eastern lineages using SPSS (IBM 2009). The eight morphological variables occupied by the two lineages were then compared one by one visually in boxplots using ggplot2 (Wickham 2009).

### Phylogeographic and Ecological Niche Analysis of Multiple Eurasian Temperate Species

In addition to the newly obtained dataset of *A*. *paludum* in the present study, we searched the Web of Science database for other phylogeographic studies on Eurasian species in order to conduct an integrated analysis. The published phylogeographic studies were filtered by the following two criteria: (1) species/subspecies or native populations of species were endemic in the regions of low and middle latitudes (1–50°N) of Eurasia, which had a similar transcontinental distribution pattern as *A*. *paludum* and (2) samples from the western and eastern regions in Eurasia were relatively balanced, and the divergence time between the lineages was estimated based on the molecular data. In total, five published studies met both criteria, including invertebrates (*A. bruennichi*, *D. magna*, *L. dispar*), birds (*P. pica*) and mammals (*M. meles*). The phylogeographic patterns and divergence times for these five species were extracted and exhibited. For the ecological niche analyses, a total of 1,645 occurrence localities of *A. paludum* based on our collection, literature records, and online database from the Global Biodiversity Information Facility online database (GBIF, http://www.gbif.org/) were used for niche modeling. The coordinates of the other five Eurasian temperate species for niche model building were directly downloaded from the GBIF database and incorporated with sites from published studies (Wu et al. 2015, 2020; Krehenwinkel et al. 2016), which determined 390 localities for *A*. *bruennichi*, 511 for *D*. *magna*, 12,982 for *L*. *dispar*, 11,256 for *P*. *pica*, and 4,229 for *M*. *meles*. We spatially thinned the occurrences using the R package spthin (Aiello-Lammens et al. 2015), resulting in a total of 212 occurrences for *A*. *paludum*, 142 for *A*. *bruennichi*, 73 for *D*. *magna*, 352 for *L*. *dispar*, 346 for *P*. *pica* and 125 for *M*. *meles*, and the nearest neighbors were no <100 km apart. Considering that a wide geographical extent of backgrounds affects the predictive power, we limited our model extent to the distributional range of the above mentioned Eurasian temperate species (i.e., Y max = 70°N, Y min = 14°N, X max = 148°E, X min = −13°E). All 19 bioclimatic variables for the current and LGM scenarios under the Community Climate System Model (CCSM4) and the Model for Interdisciplinary Research on Climate (MIROC-ESM) were downloaded from the WorldClim website (http://www.worldclim.org/). We excluded those variables with the Pearson correlation coefficient *r* > 0.7 based on a pairwise comparison of raster files in SDMTOOLBOX (Brown 2014). Five variables were retained for subsequent analysis: annual mean temperature (BIO1), mean temperature of wettest quarter (BIO8), annual precipitation (BIO12), precipitation of driest month (BIO14), and precipitation seasonality (BIO15). To predict the future potential distribution of all six Eurasian temperate species under greenhouse gas emission trajectories, two climatic scenarios, namely RCP 2.6 (the minimum greenhouse gas emission scenario) and RCP 8.5 (the maximum greenhouse gas emission scenario) for the year 2070 under CCSM4 and MIROC-ESM, were selected and downloaded from the WorldClim website. The maximum entropy method was implemented in MaxEnt 3.3.3k (Phillips et al. 2006) to develop the current distribution model. Seventy-two combinations of six feature classes (linear; linear + quadratic; hinge; linear + quadratic + hinge; linear + quadratic + hinge + product; and linear + quadratic + hinge + product + threshold) and 12 regularization multipliers (0.5, 1, 1.5, 2, 2.5, 3, 3.5, 4, 4.5, 5, 5.5, and 6) were applied to identify the optimal model parameters using the R package enmeval (Muscarella et al. 2014). The area under the curve (AUC) of the receiver operating characteristic (ROC) plot was used for model evaluation. The best current model was projected onto the set of climatic variables to infer the extent of suitable areas during the LGM and the two greenhouse gas emission scenarios under the CCSM4 and MIROC-ESM. The multivariate environmental similarity surface was used to analyze the degree of ecological change of *A*. *paludum* in the distribution area under the past and future climatic scenarios. This operation was implemented by running the “density.tools.Novel” tool in the MaxEnt. We transformed model predictions to derive binary maps under the 10th percentile training presence threshold. Thereafter, each binary layer predicted by niche models for the six Eurasian temperate species was integrated to visualize the range shifts from the LGM to the future scenarios using ArcGIS 10.1 (Environmental Systems Research Institute).

## Supplementary Material

msac089_Supplementary_Data

## Data Availability

Assembled sequences newly generated in the present study are available in GenBank (accession numbers OM849780–OM850169, OM885995–OM886384, OM929273–OM930728). Original sequence reads of genomic data are available in GenBank (accession numbers SAMN26292325–SAMN26292573, SAMN26186843–SAMN26186863).

## References

[msac089-B1] Aiello-Lammens ME, Boria RA, Radosavljevic A, Vilela B, Anderson RP. 2015. spThin: an R package for spatial thinning of species occurrence records for use in ecological niche models. Ecography 38(5):541–545.

[msac089-B2] Andersen NM . 1982. The semiaquatic bugs (Hemiptera, Gerromorpha): phylogeny, adaptations, biogeography and classification. Entomonograph 3. Klampenborg: Scandinavian Science Press Ltd.

[msac089-B3] Andersen NM . 1990. Phylogeny and taxonomy of water striders, genus *Aquarius* Schellenberg (Insecta, Hemiptera, Gerridae), with a new species from Australia. Steenstrupia 16(4):37–81.

[msac089-B4] Andersen MJ, McCullough JM, Gyllenhaal EF, Mapel XM, Haryoko T, Jønsson KA, Joseph L. 2021. Complex histories of gene flow and a mitochondrial capture event in a nonsister pair of birds. Mol Ecol. 30(9):2087–2103. 33615597 10.1111/mec.15856 PMC8252742

[msac089-B5] Andrews KR, Good JM, Miller MR, Luikart G, Hohenlohe PA. 2016. Harnessing the power of RADseq for ecological and evolutionary genomics. Nat Rev Genet. 17(2):81–92. 26729255 10.1038/nrg.2015.28 PMC4823021

[msac089-B6] Arif S, Gerth M, Hone-Millard WG, Nunes MD, Dapporto L, Shreeve TG. 2021. Evidence for multiple colonisations and Wolbachia infections shaping the genetic structure of the widespread butterfly *Polyommatus icarus* in the British Isles. Mol Ecol. 30(20):5196–5213. 34402109 10.1111/mec.16126

[msac089-B7] Avise JC, Bowen BW, Ayala FJ. 2016. In the light of evolution X: comparative phylogeography. Proc Natl Acad Sci USA. 113(29):7957–7961. 27432955 10.1073/pnas.1604338113 PMC4961136

[msac089-B8] Bolnick DI, Amarasekare P, Araújo MS, Bürger R, Levine JM, Novak M, Rudolf VHW, Schreiber SJ, Urban MC, Vasseur DA. 2011. Why intraspecific trait variation matters in community ecology. Trends Ecol Evol. 26(4):183–192. 21367482 10.1016/j.tree.2011.01.009 PMC3088364

[msac089-B9] Brown JL . 2014. SDM toolbox: a python-based GIS toolkit for landscape genetic, biogeographic and species distribution model analyses. Methods Ecol Evol. 5(7):694–700. 10.7717/peerj.4095 PMC5721907 29230356

[msac089-B10] Brown JL, Carnaval AC. 2019. A tale of two niches: methods, concepts, and evolution. Front Biogeogr. 11(4):e44158.

[msac089-B11] Capblancq T, Fitzpatrick MC, Bay RA, Exposito-Alonso M, Keller SR. 2020. Genomic prediction of (mal) adaptation across current and future climatic landscapes. Annu Rev Ecol Evol Syst. 51:245–269.

[msac089-B12] Chen PP, Nieser N, Zettel H. 2005. The aquatic and semi-aquatic bugs (Heteroptera: Nepomorpha & Gerromorpha) of Malesia. Fauna Malesiana Handbooks 5. Leiden: Koninklijke Brill NV.

[msac089-B13] Cheng L, Connor TR, Sirén J, Aanensen DM, Corander J. 2013. Hierarchical and spatially explicit clustering of DNA sequences with BAPS software. Mol Biol Evol. 30(5):1224–1228. 23408797 10.1093/molbev/mst028 PMC3670731

[msac089-B14] Cianferoni F, Mazza G. 2012. The aquatic Heteroptera (Insecta: Hemiptera) of the “Foreste Casentinesi, Monte Falterona e Campigna” National Park (Central Italy). Zootaxa 3568:36–52.

[msac089-B15] Cohen KM, Finney SC, Gibbard PL, Fan JX. 2013. The ICS international chronostratigraphic chart. Episodes 36(3):199–204.

[msac089-B16] Conesa A, Götz S, García-Gómez JM, Terol J, Talón M, Robles M. 2005. Blast2GO: a universal tool for annotation, visualization and analysis in functional genomics research. Bioinformatics 21(18):3674–3676. 16081474 10.1093/bioinformatics/bti610

[msac089-B17] Cooper A, Turney C, Hughen KA, Brook BW, McDonald HG, Bradshaw CJA. 2015. Abrupt warming events drove Late Pleistocene Holarctic megafaunal turnover. Science 349(6248):602–606. 26250679 10.1126/science.aac4315

[msac089-B18] Damgaard J, Zettel H. 2003. Genetic diversity, species phylogeny and historical biogeography of the *Aquarius paludum* group (Heteroptera: Gerridae). Insect Syst Evol. 34(3):313–327.

[msac089-B19] Dansgaard W, Johnsen SJ, Clausen HB, Dahl-Jensen D, Gundestrup NS, Hammer CU, Hvidberg CS, Steffensen JP, Sveinbjörnsdottir AE, Jouzel J, et al 1993. Evidence for general instability of past climate from a 250-kyr ice-core record. Nature 364(6434):218–220.

[msac089-B20] deMenocal P, Ortiz J, Guilderson T, Sarnthein M. 2000. Coherent high-and low-latitude climate variability during the Holocene warm period. Science 288(5474):2198–2202. 10864866 10.1126/science.288.5474.2198

[msac089-B21] Earl DA, vonHoldt BM. 2012. STRUCTURE HARVESTER: a website and program for visualizing STRUCTURE output and implementing the Evanno method. Conserv Genet Resour. 4(2):359–361.

[msac089-B22] Evanno G, Regnaut S, Goudet J. 2005. Detecting the number of clusters of individuals using the software STRUCTURE: a simulation study. Mol Ecol. 14(8):2611–2620. 15969739 10.1111/j.1365-294X.2005.02553.x

[msac089-B23] Excoffier L, Dupanloup I, Huerta-Sánchez E, Sousa VC, Foll M. 2013. Robust demographic inference from genomic and SNP data. PLOS Genet. 9(10):e1003905. 24204310 10.1371/journal.pgen.1003905 PMC3812088

[msac089-B24] Excoffier L, Lischer HE. 2010. Arlequin suite ver 3.5: a new series of programs to perform population genetics analyses under Linux and Windows. Mol Ecol Resour. 10(3):564–567. 21565059 10.1111/j.1755-0998.2010.02847.x

[msac089-B25] Favre A, Päckert M, Pauls SU, Jähnig SC, Uhl D, Michalak I, Muellner-Riehl AN. 2015. The role of the uplift of the Qinghai-Tibetan Plateau for the evolution of Tibetan biotas. Biol Rev. 90(1):236–253. 24784793 10.1111/brv.12107

[msac089-B26] Fedorov VB, Trucchi E, Goropashnaya AV, Waltari E, Whidden SE, Stenseth NC. 2020. Impact of past climate warming on genomic diversity and demographic history of collared lemmings across the Eurasian Arctic. Proc Natl Acad Sci U S A. 117(6):3026–3033. 31988125 10.1073/pnas.1913596117 PMC7022146

[msac089-B27] Fields PD, Obbard DJ, McTaggart SJ, Galimov Y, Little TJ, Ebert D. 2018. Mitogenome phylogeographic analysis of a planktonic crustacean. Mol Phylogenet Evol. 129:138–148. 29920335 10.1016/j.ympev.2018.06.028

[msac089-B28] Foll M, Gaggiotti O. 2008. A genome-scan method to identify selected loci appropriate for both dominant and codominant markers: a Bayesian perspective. Genetics 180(2):977–993. 18780740 10.1534/genetics.108.092221 PMC2567396

[msac089-B29] Fossøy F, Sorenson MD, Liang W, Ekrem T, Moksnes A, Møller AP, Rutila J, Røskaft E, Takasu F, Yang CC, et al 2016. Ancient origin and maternal inheritance of blue cuckoo eggs. Nat Commun. 7(1):1–6. 10.1038/ncomms10272 PMC4729921 26754355

[msac089-B30] Frichot E, François O. 2015. LEA: an R package for landscape and ecological association studies. Methods Ecol Evol. 6(8):925–929.

[msac089-B31] Gottfried M, Pauli H, Futschik A, Akhalkatsi M, Barančok P, Alonso JLB, Coldea G, Dick J, Erschbamer B, Calzado MRF, et al 2012. Continent-wide response of mountain vegetation to climate change. Nat Clim Change 2(2):111–115.

[msac089-B32] Harada T, Shiraki T, Takenaka S, Sekimoto T, Emi K, Furutani T. 2013. Change in reproductive and dispersal traits in the water strider, *Aquarius paludum* (Fabricius) and global warming. Nat Sci. 5(1A):156–162.

[msac089-B33] Head MJ, Gibbard PL. 2005. Early-Middle Pleistocene transitions: an overview and recommendation for the defining boundary. J Geol Soc Lond. 247(1):1–18.

[msac089-B34] Heede R . 2014. Tracing anthropogenic carbon dioxide and methane emissions to fossil fuel and cement producers, 1854–2010. Clim Change 122(1):229–241.

[msac089-B35] Hinojosa JC, Koubínová D, Szenteczki MA, Pitteloud C, Dincă V, Alvarez N, Vila R. 2019. A mirage of cryptic species: genomics uncover striking mitonuclear discordance in the butterfly *Thymelicus sylvestris*. Mol Ecol. 28(17):3857–3868. 31233646 10.1111/mec.15153

[msac089-B36] Hou Z, Sket B, Fišer C, Li S. 2011. Eocene habitat shift from saline to freshwater promoted Tethyan amphipod diversification. Proc Natl Acad Sci U S A. 108(35):14533–14538. 21844362 10.1073/pnas.1104636108 PMC3167504

[msac089-B37] Huang ZG, Zhang WQ, Jiang LM. 2005. The characteristics of Quaternary climate fluctuation in the tropics of China. Cartogr Geogr Inf Sci. 21(4):65–70.

[msac089-B38] Jakubas D, Wojczulanis-Jakubas K, Iliszko LM, Strøm H, Stempniewicz L. 2017. Habitat foraging niche of a High Arctic zooplanktivorous seabird in a changing environment. Sci Rep. 7:16203. 29176574 10.1038/s41598-017-16589-7 PMC5701252

[msac089-B39] Jombart T . 2008. adegenet: a R package for the multivariate analysis of genetic markers. Bioinformatics 24(11):1403–1405. 18397895 10.1093/bioinformatics/btn129

[msac089-B40] Keightley PD, Trivedi U, Thomson M, Oliver F, Kumar S, Blaxter ML. 2009. Analysis of the genome sequences of three *Drosophila melanogaster* spontaneous accumulation lines. Genome Res. 19(7):1195–1201. 19439516 10.1101/gr.091231.109 PMC2704435

[msac089-B41] Koshkarova VL, Koshkarov AD. 2004. Regional signatures of changing landscape and climate of northern central Siberia in the Holocene. Russ Geol Geophys. 45(6):672–685.

[msac089-B42] Krehenwinkel H, Graze M, Rödder D, Tanaka K, Baba YG, Muster C, Uhl G. 2016. A phylogeographical survey of a highly dispersive spider reveals eastern Asia as a major glacial refugium for Palaearctic fauna. J Biogeogr 43(8):1583–1594.

[msac089-B43] Lorenzen ED, Nogués-Bravo D, Orlando L, Weinstock J, Binladen J, Marske KA, Ugan A, Borregaard MK, Gilbert MTP, Nielsen R, et al 2011. Species-specific responses of late quaternary megafauna to climate and humans. Nature 479:359–364. 22048313 10.1038/nature10574 PMC4070744

[msac089-B44] Louis M, Skovrind M, Castruita JAS, Garilao C, Kaschner K, Gopalakrishnan S, Haile JS, Lydersen C, Kovacs KM, Garde E, et al 2020. Influence of past climate change on phylogeography and demographic history of narwhals, Monodon monoceros. Proc R Soc B. 287(1925):20192964. 10.1098/rspb.2019.2964 PMC7211449 32315590

[msac089-B45] Maguilla E, Escudero M, Hipp AL, Luceño M. 2017. Allopatric speciation despite historical gene flow: divergence and hybridization in *Carex furva* and *C. lucennoiberica* (Cyperaceae) inferred from plastid and nuclear RAD-seq data. Mol Ecol. 26(20):5646–5662. 28742230 10.1111/mec.14253

[msac089-B46] Marmi J, Lopez-Giraldez F, Macdonald DW, Calafell F, Zholnerovskaya E, Domingo-Roura X. 2006. Mitochondrial DNA reveals a strong phylogeographic structure in the badger across Eurasia. Mol Ecol. 15(4):1007–1020. 16599963 10.1111/j.1365-294X.2006.02747.x

[msac089-B47] McGaughran A, Laver R, Fraser C. 2021. Evolutionary responses to warming. Trends Ecol Evol. 36(7):591–600. 33726946 10.1016/j.tree.2021.02.014

[msac089-B48] Muscarella R, Galante PJ, Soley-Guardia M, Boria RA, Kass JM, Uriarte M, Anderson RP. 2014. ENMeval: an R package for conducting spatially independent evaluations and estimating optimal model complexity for Maxent ecological niche models. Methods Ecol Evol. 5(11):1198–1205.

[msac089-B49] Nelson RM, Wallberg A, Simões ZLP, Lawson DJ, Webster MT. 2017. Genomewide analysis of admixture and adaptation in the Africanized honeybee. Mol Ecol. 26(14):3603–3617. 28378497 10.1111/mec.14122

[msac089-B50] Omann I, Stocker A, Jäger J. 2009. Climate change as a threat to biodiversity: an application of the DPSIR approach. Ecol Econ. 69(1):24–31.

[msac089-B51] Palombo MR, Raia P, Giovinazzo C. 2005. Early-Middle Pleistocene structural changes in mammalian communities from the Italian peninsula. Geol Soc Lond Spec Pub. 247(1):251–262.

[msac089-B52] Peterson BK, Weber JN, Kay EH, Fisher HS, Hoekstra HE. 2012. Double digest RADseq: an inexpensive method for de novo SNP discovery and genotyping in model and non-model species. PLoS One 7(5):e37135. 22675423 10.1371/journal.pone.0037135 PMC3365034

[msac089-B53] Phillips SJ, Anderson RP, Schapire RE. 2006. Maximum entropy modeling of species geographic distributions. Ecol Model. 190(3–4):231–259.

[msac089-B54] Platt DE, Haber M, Dagher-Kharrat MB, Douaihy B, Khazen G, Bonab MA, Salloum A, Mouzaya F, Luiselli D, Tyler-Smith C, et al 2017. Mapping post-glacial expansions: the peopling of Southwest Asia. Sci Rep. 7:40338. 28059138 10.1038/srep40338 PMC5216412

[msac089-B55] Pritchard JK, Stephens M, Donnelly P. 2000. Inference of population structure using multilocus genotype data. Genetics 155(2):945–959. 10835412 10.1093/genetics/155.2.945 PMC1461096

[msac089-B56] Privé F, Luu K, Vilhjálmsson BJ, Blum MG. 2020. Performing highly efficient genome scans for local adaptation with R package pcadapt version 4. Mol Biol Evol. 37(7):2153–2154. 32343802 10.1093/molbev/msaa053

[msac089-B57] Rögl F . 1998. Palaeogeographic considerations for Mediterranean and Paratethys seaways (Oligocene to Miocene). Ann Naturhis Mus Wien. 99A:279–310.

[msac089-B58] Roycroft EJ, Le Port A, Lavery SD. 2019. Population structure and male-biased dispersal in the short-tail stingray *Bathytoshia brevicaudata* (Myliobatoidei: Dasyatidae). Conserv Genet. 20(4):717–728.

[msac089-B59] Song G, Zhang RY, Alström P, Irestedt M, Cai TL, Qu YH, Ericson PGP, Fjeldså J, Lei FM. 2018. Complete taxon sampling of the avian genus Pica (magpies) reveals ancient relictual populations and synchronous Late-Pleistocene demographic expansion across the Northern Hemisphere. J Avian Biol. 49 (2):jav-01612.

[msac089-B60] Steffen W, Rockström J, Richardson K, Lenton TM, Folke C, Liverman D, Summerhayes CP, Barnosky AD, Cornell SE, Crucifix M, et al 2018. Trajectories of the earth system in the Anthropocene. Proc Natl Acad Sci U S A. 115(33):8252–8259. 30082409 10.1073/pnas.1810141115 PMC6099852

[msac089-B61] Theodoridis S, Patsiou TS, Randin C, Conti E. 2018. Forecasting range shifts of a cold-adapted species under climate change: are genomic and ecological diversity within species crucial for future resilience? Ecography 41(8):1357–1369.

[msac089-B62] Usoltsev VA, Chen B, Shobairi SOR, Tsepordey IS, Chasovskikh VP, Anees SA. 2020. Patterns for *Populus* spp. stand biomass in gradients of winter temperature and precipitation of Eurasia. Forests 11(9):906.

[msac089-B63] Wang R, Abelmann A, Li B, Zhao Q. 2000. Abrupt variations of the radiolarian fauna at Mid-Pleistocene climate transition in the South China Sea. Chi Sci Bull. 45(10):952–955.

[msac089-B64] Wang F, Xiong Z, Dai X, Li Y, Wang L. 2020. The response of the species diversity pattern of *Populus* to climate change in China. Phys Chem Earth. 116:102858.

[msac089-B65] Wickham H . 2009. Ggplot2: elegant graphics for data analysis. 2nd ed. New York (NY): Springer.

[msac089-B66] Willeit M, Ganopolski A, Calov R, Brovkin V. 2019. Mid-Pleistocene transition in glacial cycles explained by declining CO_2_ and regolith removal. Nat Rev Genet. 5(4):eaav7337. 10.1126/sciadv.aav7337 PMC6447376 30949580

[msac089-B67] Wu Y, Bogdanowicz SM, Andres JA, Vieira KA, Wang B, Cossé A, Pfister SE. 2020. Tracking invasions of a destructive defoliator, the gypsy moth (Erebidae: *Lymantria dispar*): population structure, origin of intercepted specimens, and Asian introgression into North America. Evol Appl. 13(8):2056–2070. 32908604 10.1111/eva.12962 PMC7463338

[msac089-B68] Wu YK, Molongoski JJ, Winograd DF, Bogdanowicz SM, Louyakis AS, Lance DR, Mastro VC, Harrison RG. 2015. Genetic structure, admixture and invasion success in a Holarctic defoliator, the gypsy moth (*Lymantria dispar*. Lepidoptera: Erebidae). Mol Ecol. 24(6):1275–1291. 25655667 10.1111/mec.13103

[msac089-B69] Ye Z, Chen DY, Yuan JJ, Zheng CG, Yang X, Wang WW, Zhang YY, Wang SQ, Kun J, Bu WJ. 2020. Are population isolations and declines a threat to island endemic water striders? A lesson from demographic and niche modelling of *Metrocoris esakii* (Hemiptera: Gerridae). Mol Ecol. 29(23):4573–4587. 33006793 10.1111/mec.15669

[msac089-B70] Ye J, Fang L, Zheng HK, Zhang Y, Chen J, Zhang ZJ, Wang J, Li S, Li RQ, Bolund L, et al 2006. WEGO: a web tool for plotting GO annotations. Nucleic Acids Res. 34:W293–W297. 16845012 10.1093/nar/gkl031 PMC1538768

[msac089-B71] Ye Z, Yuan JJ, Zhen YH, Damgaard J, Yamada K, Zhu XX, Jiang K, Yang X, Wang WW, Wang SJ, et al 2020. Local environmental selection and lineage admixture act as significant mechanisms in the adaptation of the widespread East Asian pond skater *Gerris latiabdominis* to heterogeneous landscapes. J Biogeogr. 47(5):1154–1165.

[msac089-B72] Zachos J, Pagani M, Sloan L, Thomas E, Billups K. 2001. Trends, rhythms, and aberrations in global climate 65 Ma to present. Science 292(5517):686–693. 11326091 10.1126/science.1059412

