# Supplementary material for: Climate Warming Since the Holocene Accelerates West–East Communication for the Eurasian Temperate Water Strider Species Aquarius paludum
Source: Mol Biol Evol. 2022 Apr 28;39(5):msac089. doi: 10.1093/molbev/msac089 (PMC9087890; doi:10.1093/molbev/msac089)
Supplement: msac089_Supplementary_Data [file msac089_supplementary_data.zip › Supplementary_figures_S1-S21,_tables_S5-S9_and_Supplementary_methods_final_version.pdf]

Supplementary figures

Supplementary fig. S1 Schematics of the 10 demographic models compared in this study. Arrows represent the presence of gene flow and abbreviations (W and E) correspond to western and eastern lineages of *A. paludum* as defined in population structure analyses. Detailed descriptions of the models M1 to M10 can be found in the Supplementary table S7.

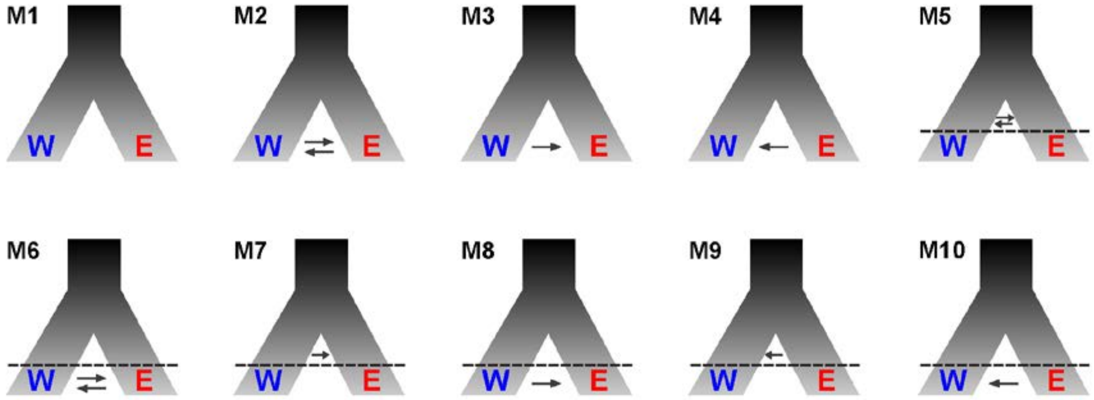

Supplementary fig. S2 Putative outliers identified by Bayescan analysis. Locus-specific F_{ST} coefficient is plotted against log10 (q value). The vertical solid line represents the threshold for being under selection after correction with FDR (0.05). 74 SNP coloured in red is probably under divergent selection, and 40 SNPs coloured in blue are probably under balancing selection.

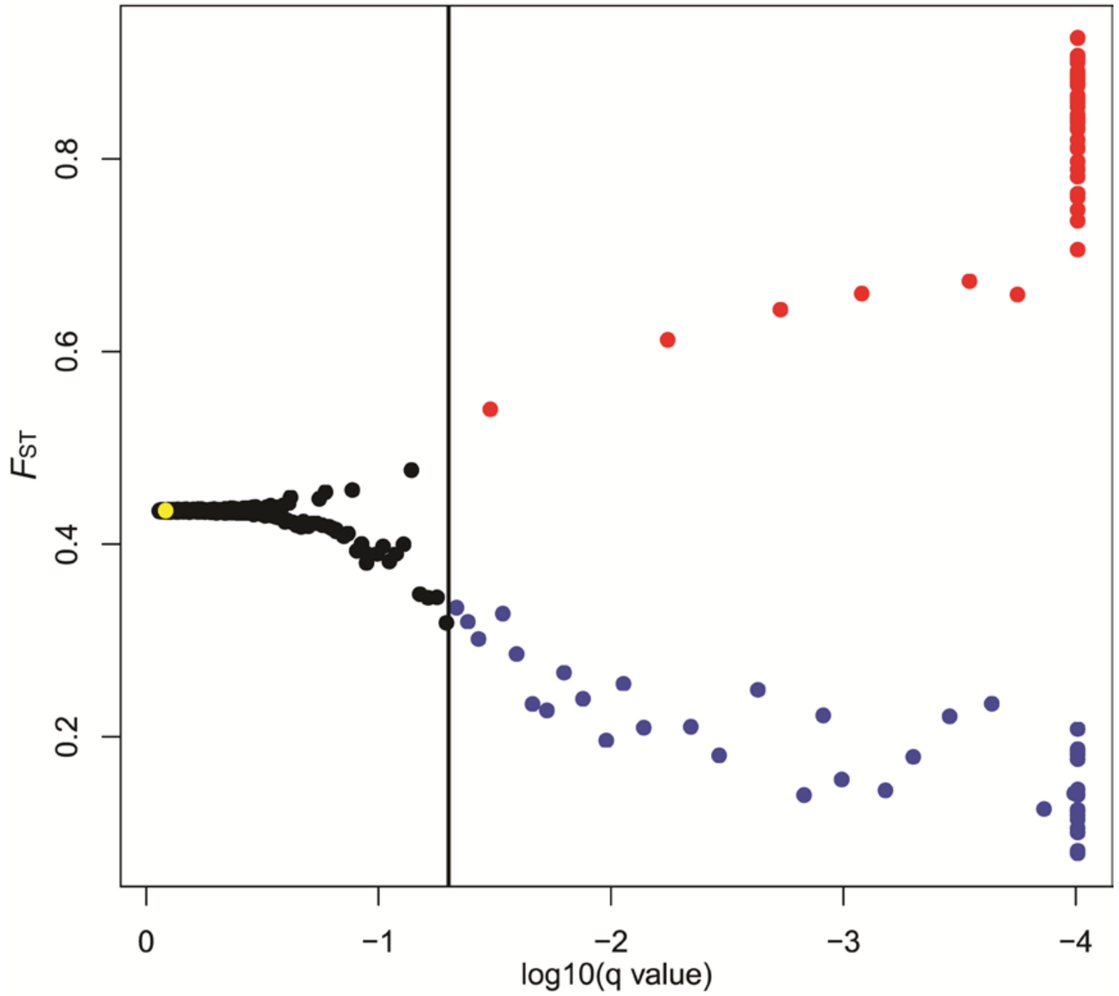

Supplementary fig. S3 Population structure estimated by the snmf method for K=2 based on the 1,105 unlinked SNPs of the ddRAD_95 USNPs dataset. Western lineage = blue strips; Eastern lineage = red strips.

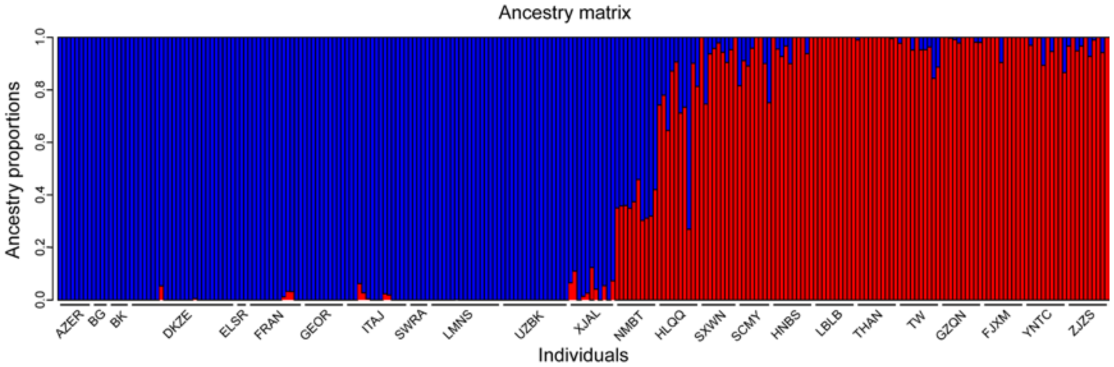

Supplementary fig. S4 Population structures estimated by the DAPC method for K=2 based on the 1,105 unlinked SNPs of the ddRAD_95 USNPs dataset. Western lineage = blue dots; Eastern lineage = red dots; hybrid populations = purple dots.

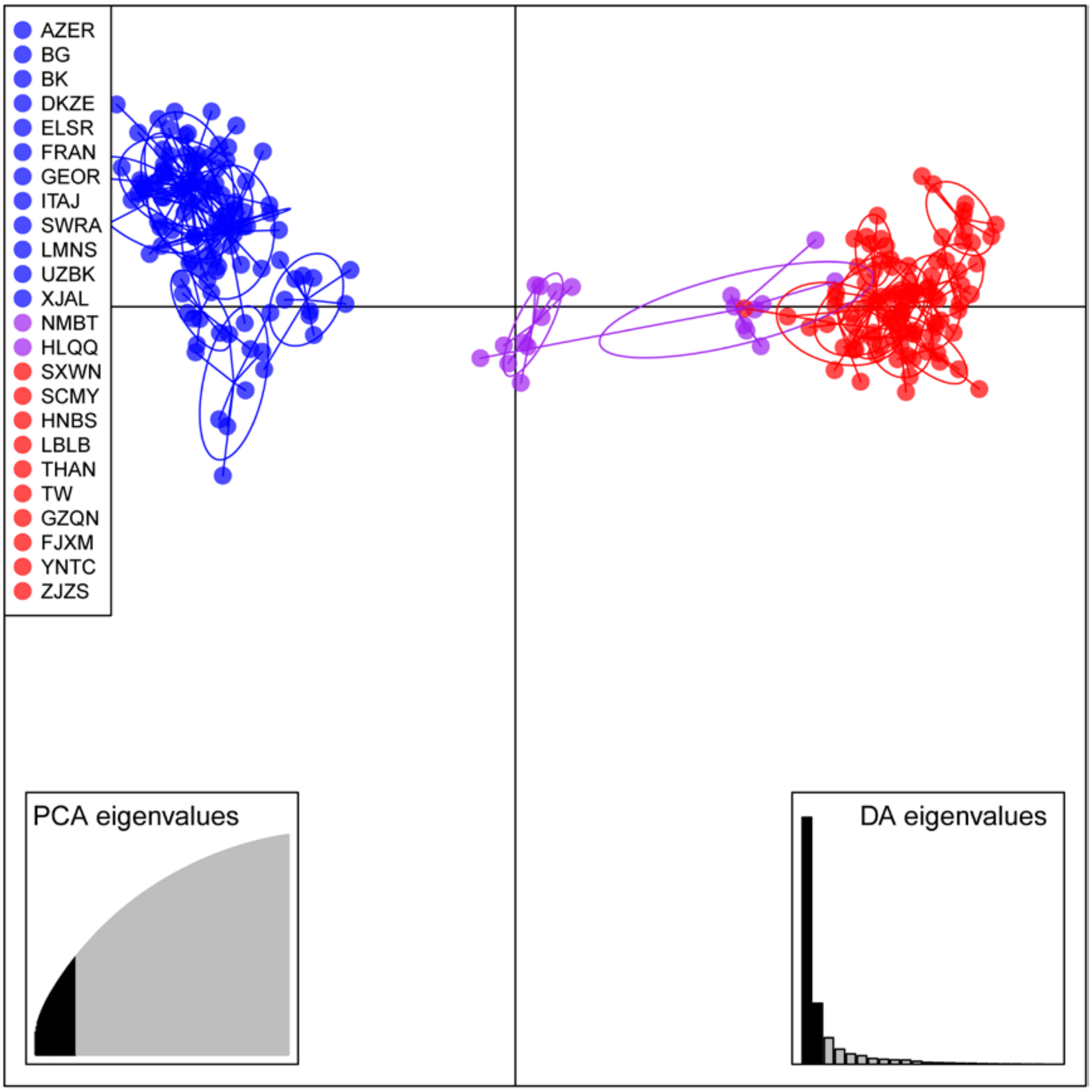

Supplementary fig. S5 Population structures estimated by the STRUCTURE method within the respective lineages. (a) the western lineage (showing K values from 2 to 4, optimal K = 3); (b) the eastern lineage (showing K values from 2 to 4, optimal K = 2).

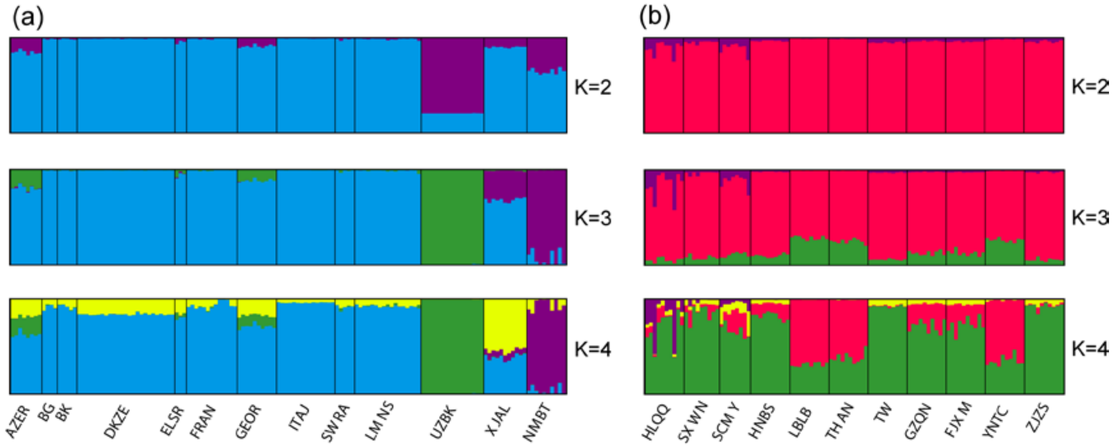

Supplementary fig. S6 Population structures estimated by the BAPS method for K=2 of *A. paludum* throughout the Eurasia. (a) result based on concatenated mitochondrial genes (COI+COII); (b) result based on 13 PCGs of mitochondrial genome. Western lineage = blue pieces; Eastern lineage = red pieces. Asterisk pieces represent hybrid populations inferred by the nuclear data.

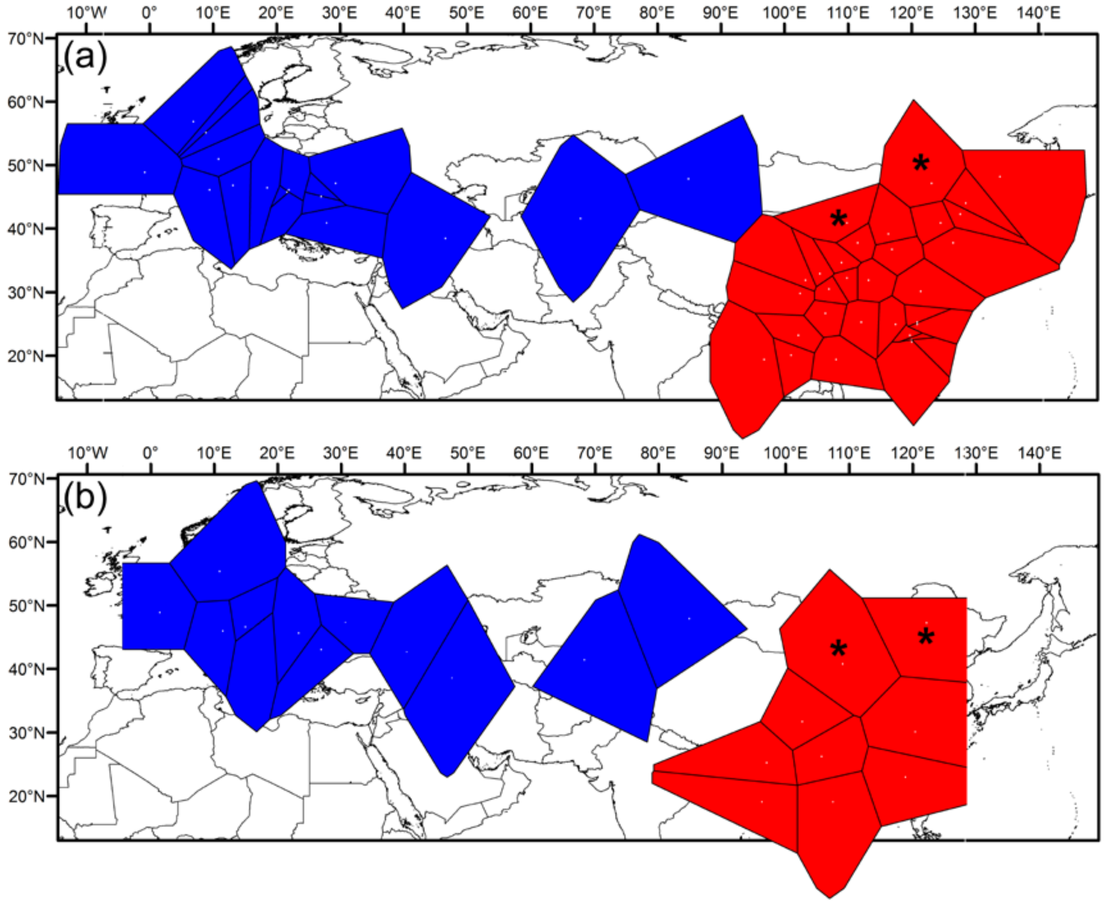

Supplementary fig. S7 Scatter plots showing the relationship between the genetic distances $F_{ST}/(1 - F_{ST})$ and geographical/environmental/resistance-climatic distances among the populations within *A. paludum*. (a) isolation by distance (IBD); (b) isolation by environment (IBE); (c) isolation by resistance-climate (IBR).

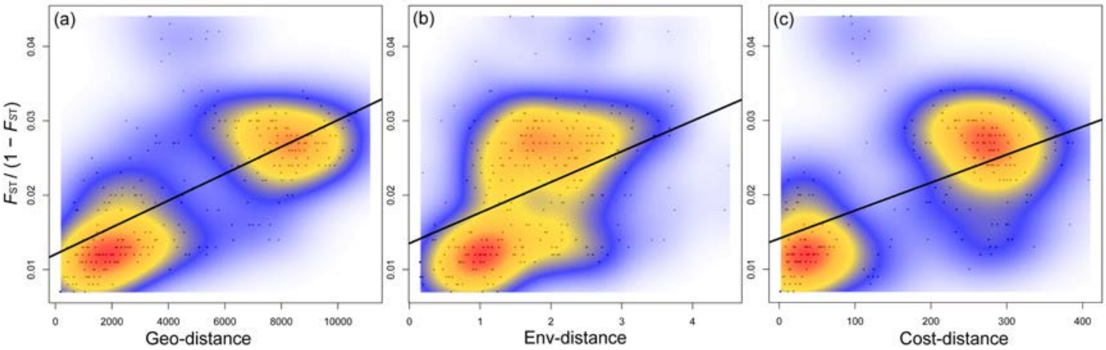

Supplementary fig. S8 Least cost distances (LCDs) used for visualizing dispersal corridors of *A. paludum* across the landscapes (habitat heterogeneity) for the two periods. Regions with lower values indicate putatively higher resistances to movement. Population locations used for ddRAD-seq analyses are shown as red dots. The gray line between populations depicts the least-cost path. A potential northern corridor is exhibited.

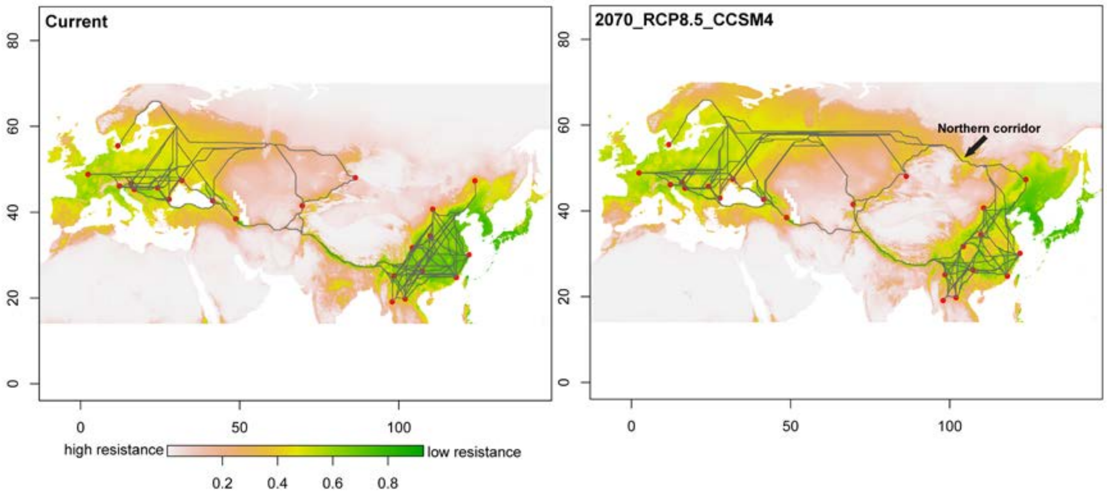

Supplementary fig. S9 Bayesian skyline plot (BSP) based on mitochondrial COI sequences showing effective population size changes through time for the two genetic lineages of *A. paludum*. Estimates of means are joined by a solid thick line while the thin lines delineate the 95% confidence intervals limits.

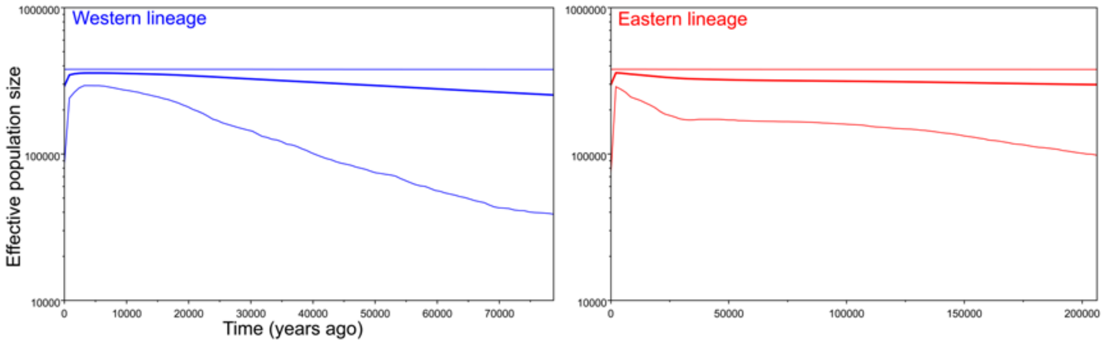

Supplementary fig. S10 (a) Manhattan plots of the results obtained with pcadapt. (b) Venn diagrams showing the 5610 outlier SNPs using pcadapt to detect local adaptation by using three cutoff methods (i.e., q-values, Benjamini-Hochberg Procedure and Bonferroni correction). The numbers and percentages of overlapping outlier SNPs among the different approaches are also illustrated. (c) Gene ontology annotations of the candidate genes under potential local selection in *A. paludum*. Three categories: Cellular Component, Molecular Function and Biological Process, are used to visualize the potential functions of enriched genes.

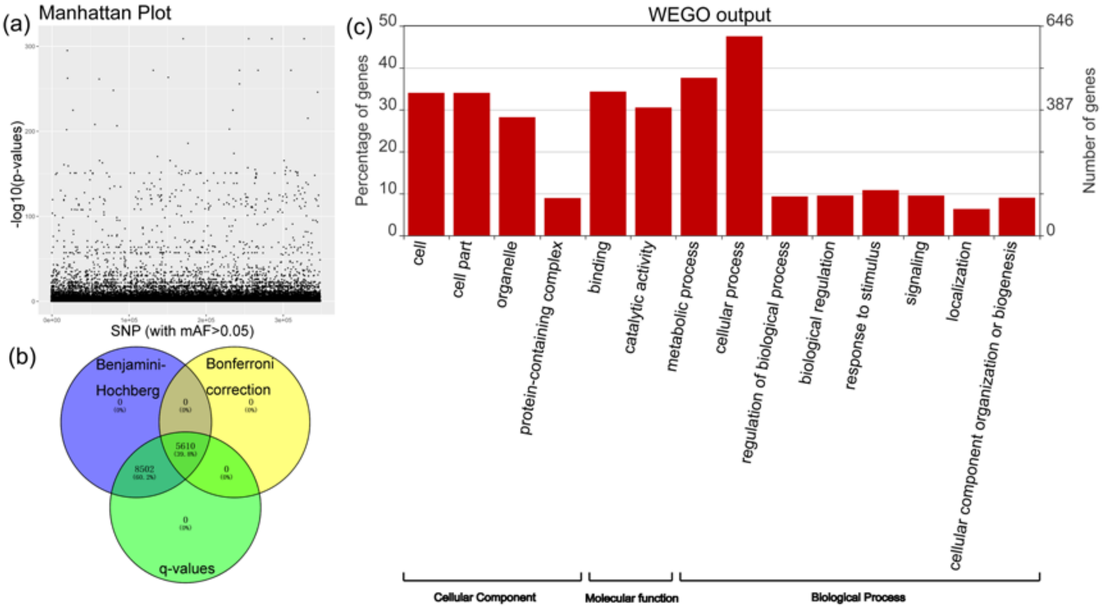

Supplementary fig. S11 Principal component analysis (PCA) of eight morphological variables associated with 233 specimens of the western lineage and eastern lineage within *A. paludum*.

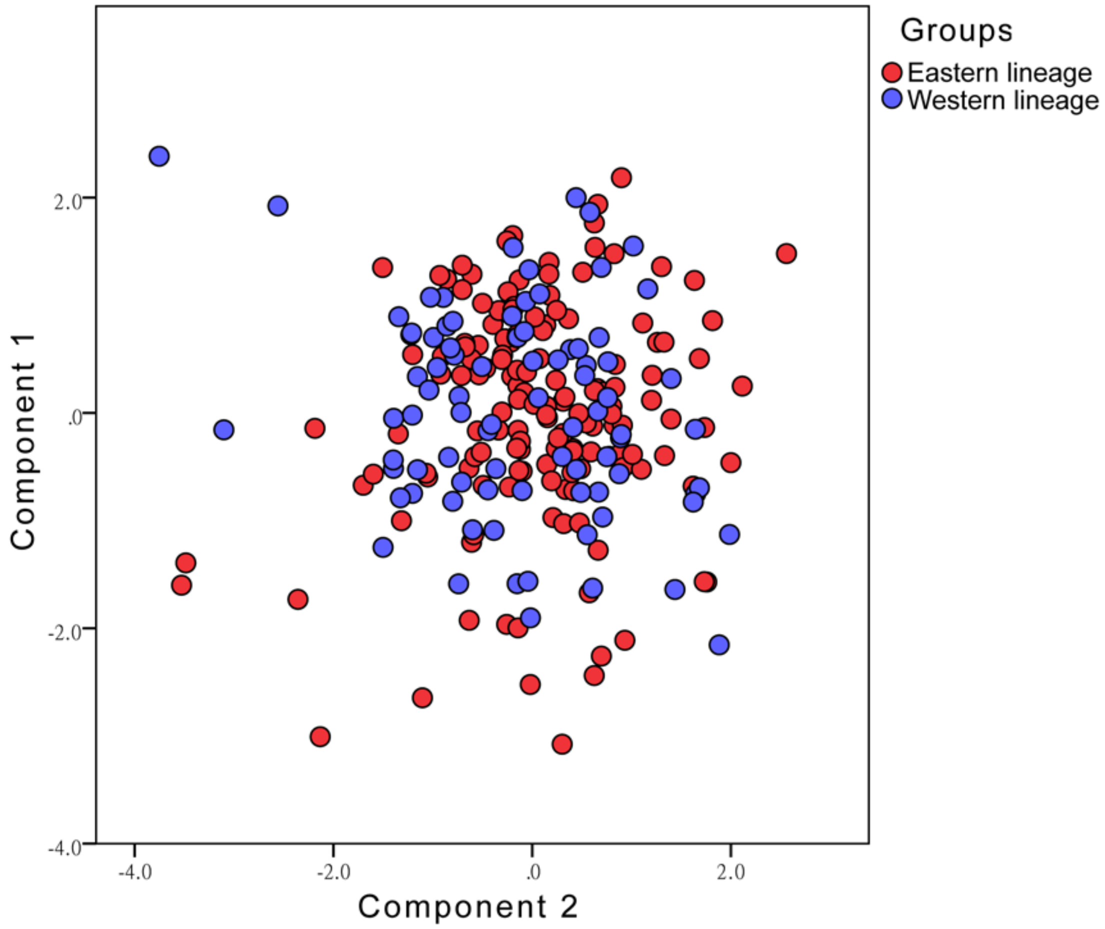

Supplementary fig. S12 Boxplots of the eight diagnostic characters (body length, body width, head length, pronotum length, hind femur length, abdomen length, seventh abdominal sternum length and connexival spine) comparing between western and eastern lineages of *A. paludum*. Values significant at $p < 0.05$ and $p < 0.01$ with the one and two asterisks, respectively. NS = none significant.

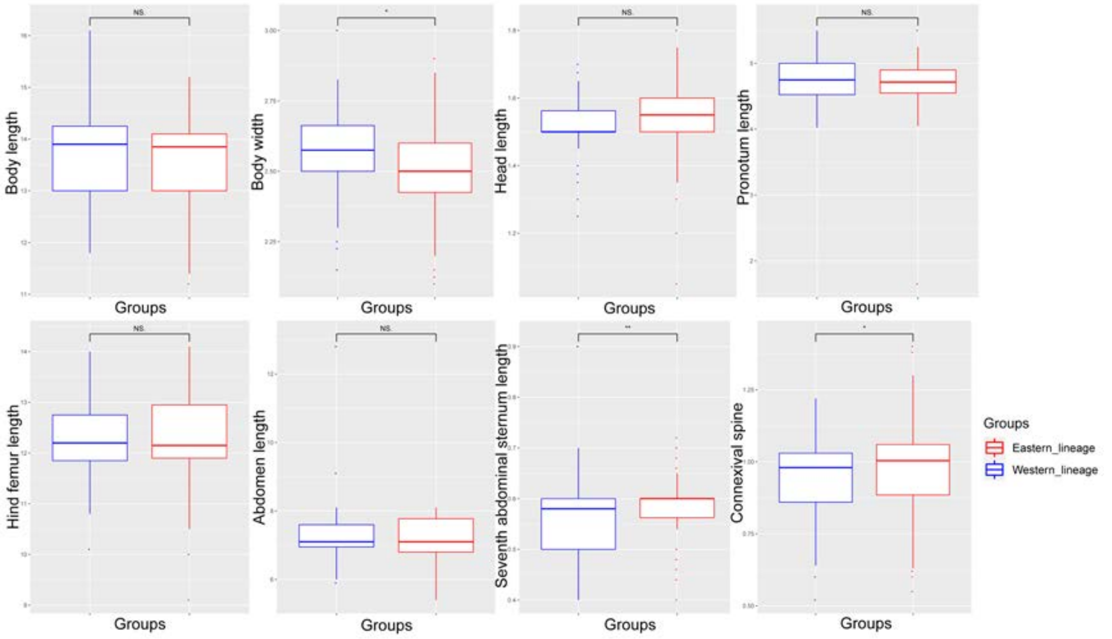

Supplementary fig. S13 Putative phylogeographic structures and divergence times between the western and eastern populations for the five Eurasian species, which were copied from published studies (Krehenwinkel et al. 2016; Fields et al. 2018; Wu et al. 2015; Marmi et al. 2006; Song et al. 2018).

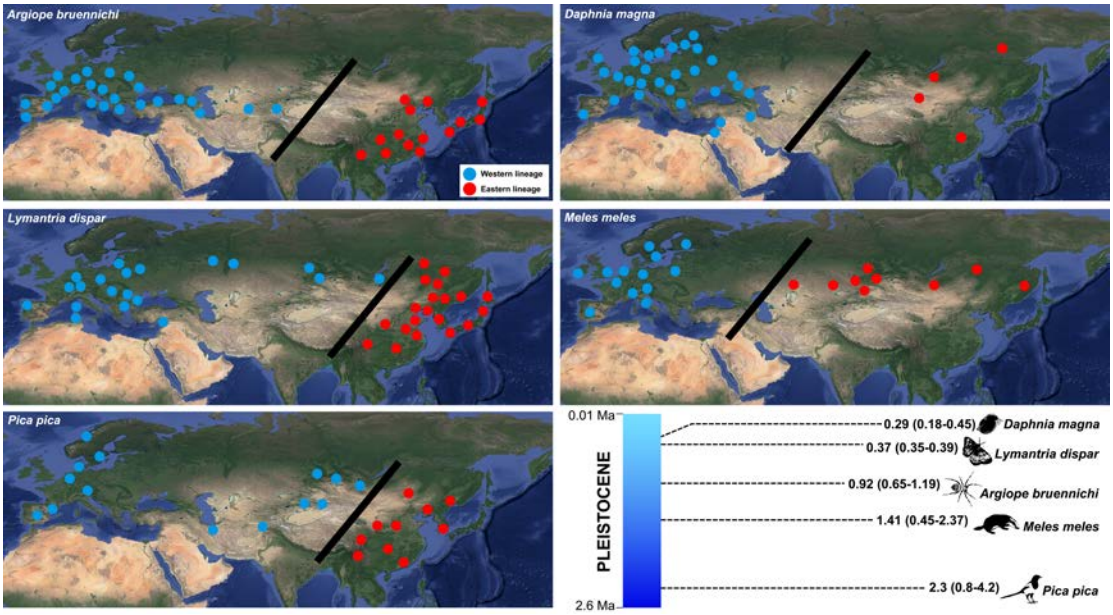

Supplementary fig. S14 Performances of niche model of the six Eurasian temperate species under different settings. (a) *Aquarius paludum*; (b) *Argiope bruennichi*; (c) *Daphnia magna*; (d) *Lymantria dispar*; (e) *Meles meles*; (f) *Pica pica*. Red arrow indicates the delta AICc-chosen setting. L= Linear; Q = Quadratic; H = Hinge; P = Product; T = Threshold.

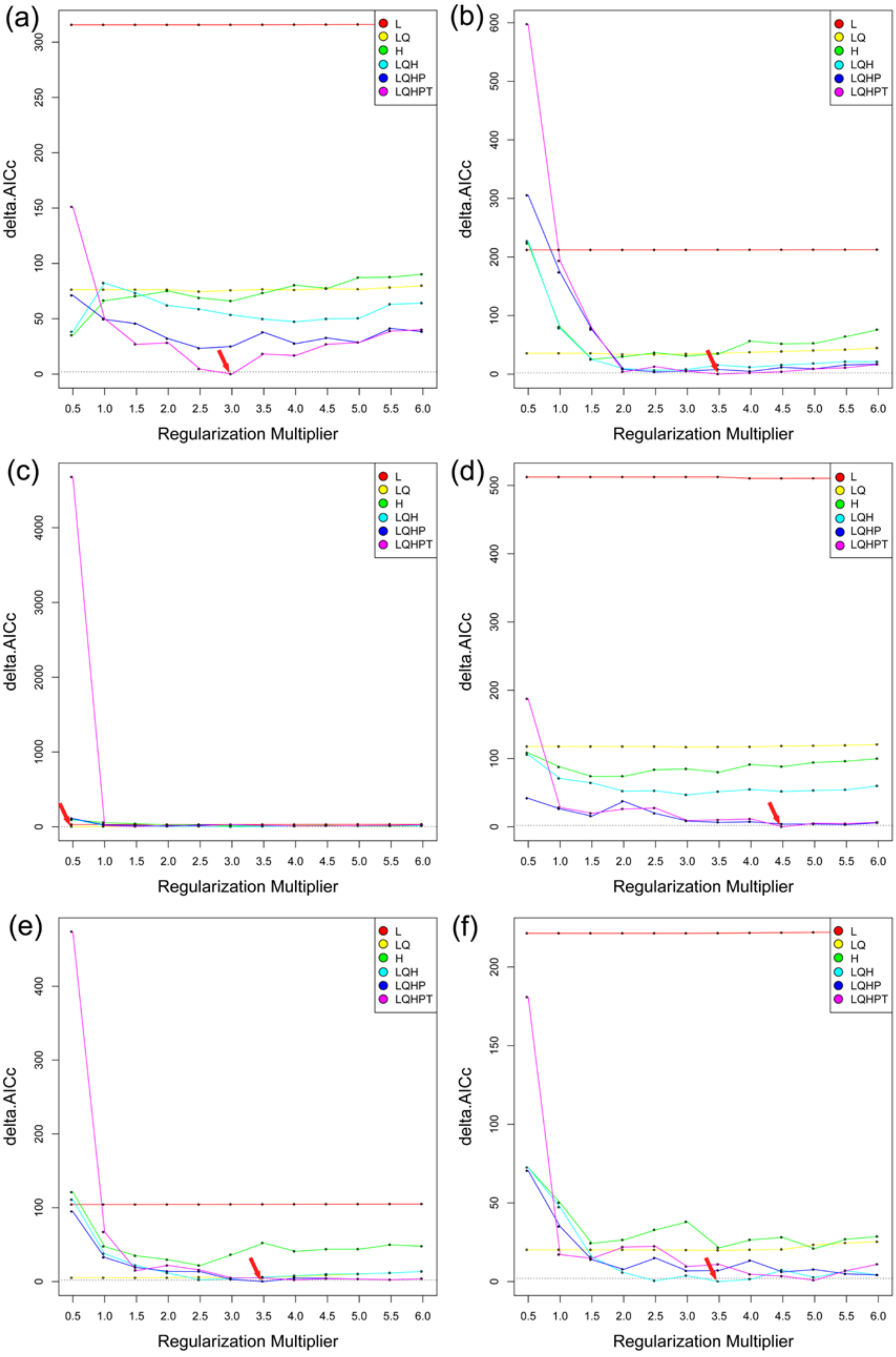

Supplementary fig. S15 Multivariate environmental similarity surface (MESS) for *Aquarius paludum* under past and future extreme climate change scenarios. (a) LGM_CCSM4; (b) LGM_MIROC-ESM; (c) 2070_RCP2.6_CCSM4; (d) 2070_RCP8.5_CCSM4; (e) 2070_RCP2.6_MIROC-ESM; (f) 2070_RCP8.5_MIROC-ESM.

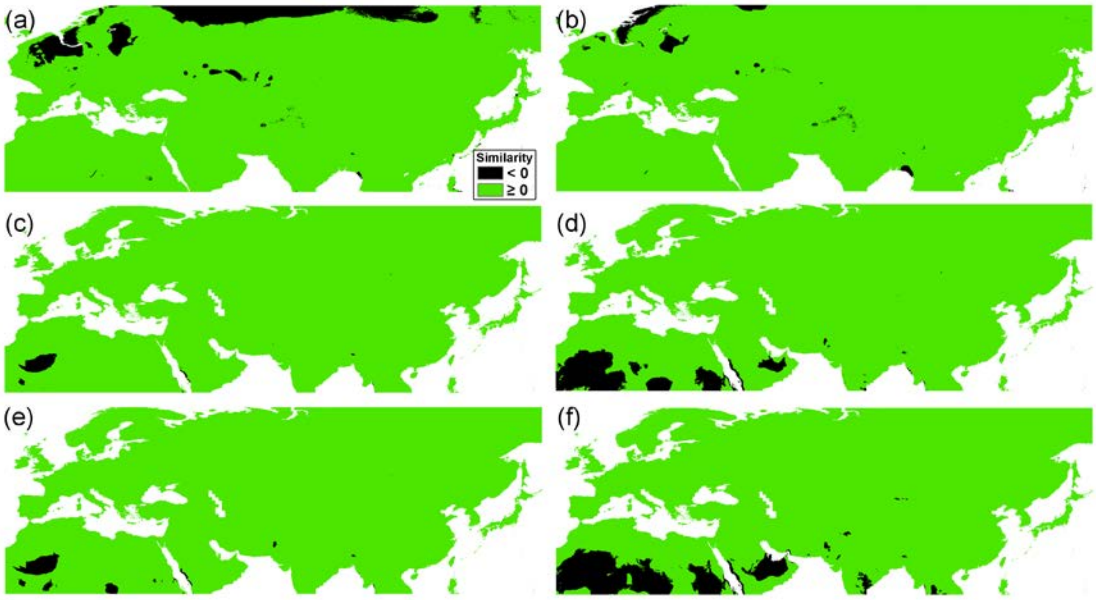

Supplementary fig. S16 Integrated modelled suitable areas of six Eurasian temperate species (*Aquarius paludum*, *Argiope bruennichi*, *Daphnia magna*, *Lymantria dispar*, *Pica pica* and *Meles meles*) under the 10 percentile training presence threshold from the current climatic condition, the LGM and the maximum greenhouse gas emission scenario (RCP 8.5) for the year 2070 under the CCSM4. Occurrence localities (green dots) of the six Eurasian temperate species are used for ecological niche modelling. A potential northern corridor is shown in the 2070_RCP8.5 predicted areas.

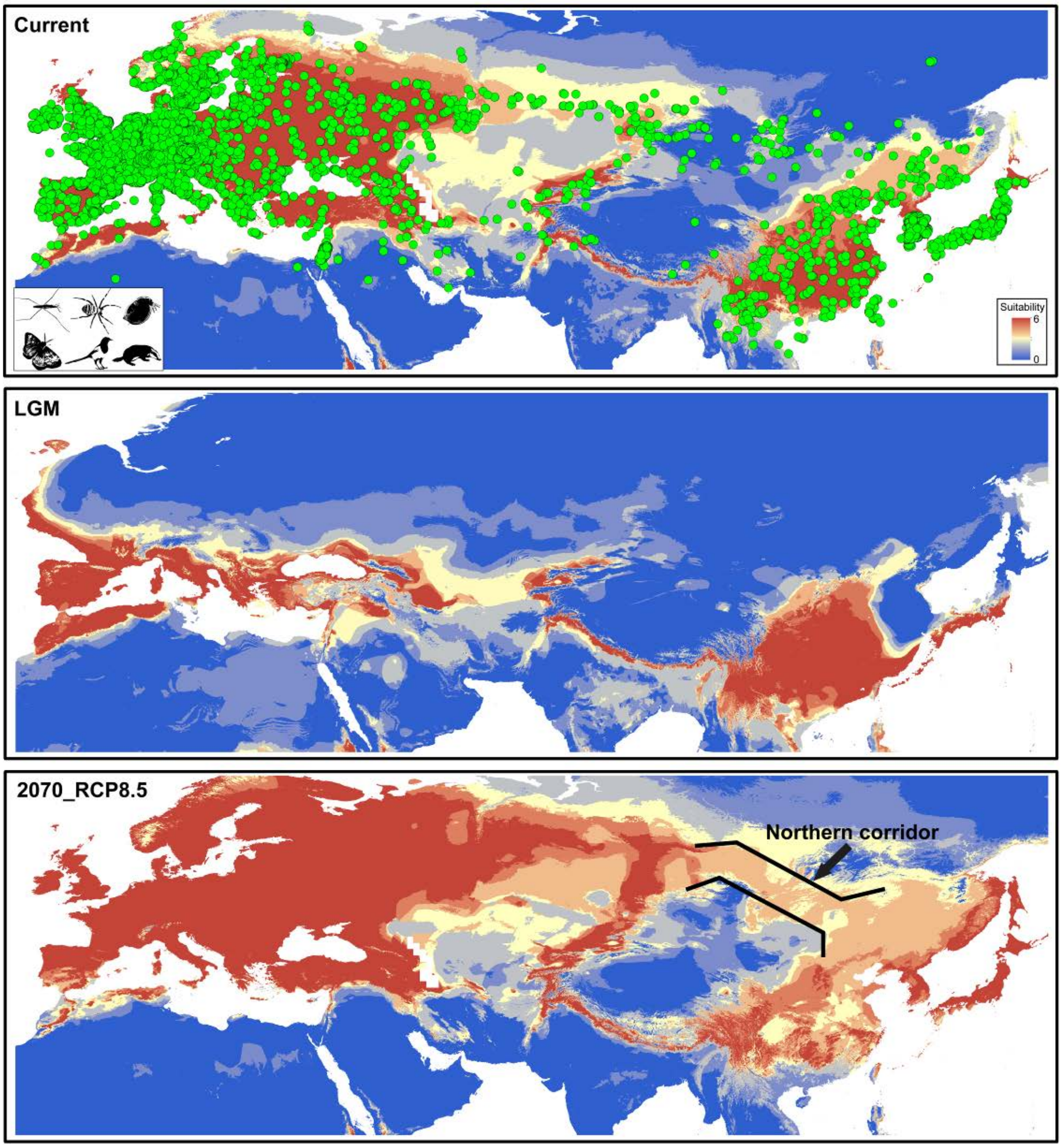

Supplementary fig. S17 Modelled suitable areas of *Argiope bruennichi* throughout the Eurasia under the 10 percentile training presence threshold from the current climatic condition, the LGM and the two greenhouse gas emission scenarios (RCP 2.6 and RCP 8.5) for the year 2070 under the CCSM4 and MIROC-ESM. Red colours indicate habitat suitability areas. Occurrence localities (yellow dots) are used for ecological niche modelling.

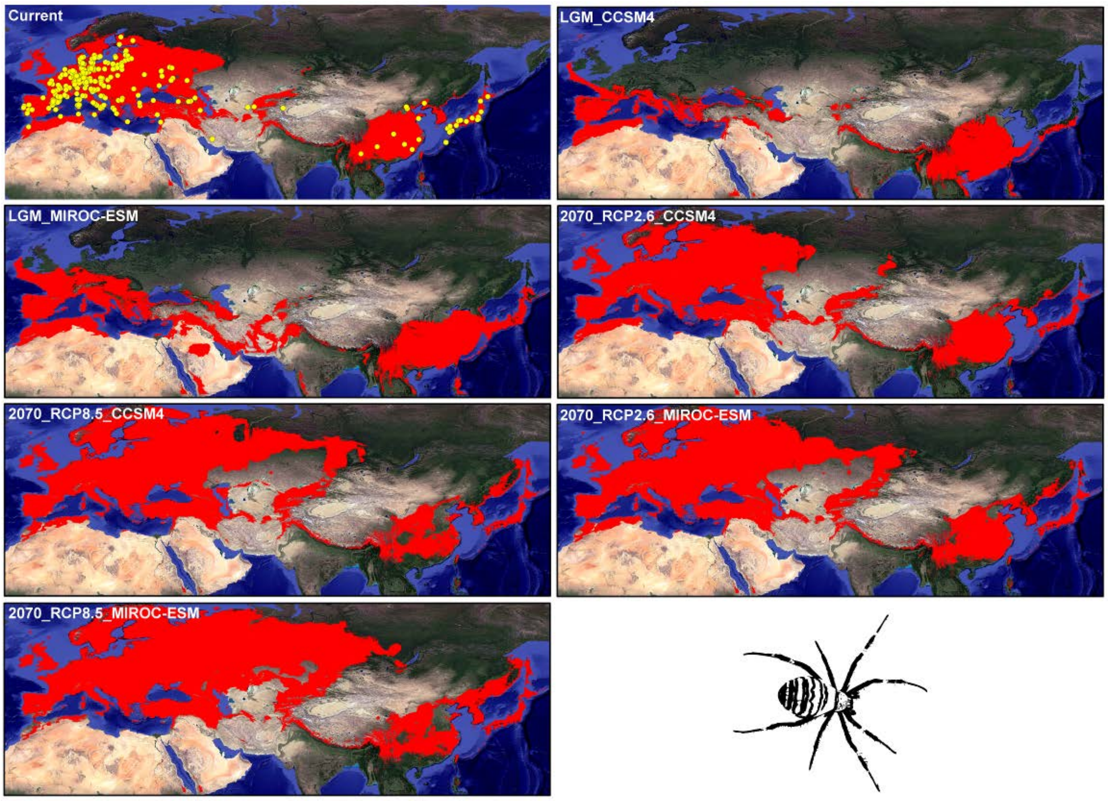

Supplementary fig. S18 Modelled suitable areas of *Daphnia magna* throughout the Eurasia under the 10 percentile training presence threshold from the current climatic condition, the LGM and the two greenhouse gas emission scenarios (RCP 2.6 and RCP 8.5) for the year 2070 under the CCSM4 and MIROC-ESM. Red colours indicate habitat suitability areas. Occurrence localities (yellow dots) are used for ecological niche modelling.

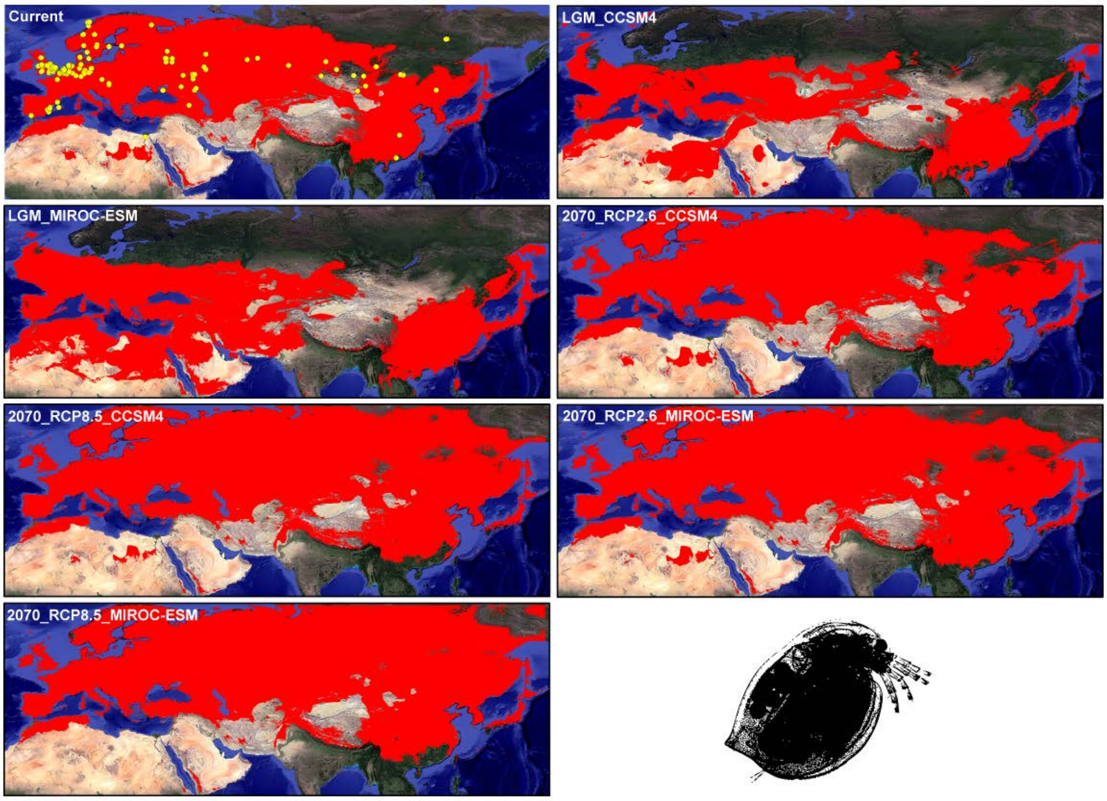

Supplementary fig. S19 Modelled suitable areas of *Lymantria dispar* throughout the Eurasia under the 10 percentile training presence threshold from the current climatic condition, the LGM and the two greenhouse gas emission scenarios (RCP 2.6 and RCP 8.5) for the year 2070 under the CCSM4 and MIROC-ESM. Red colours indicate habitat suitability areas. Occurrence localities (yellow dots) are used for ecological niche modelling.

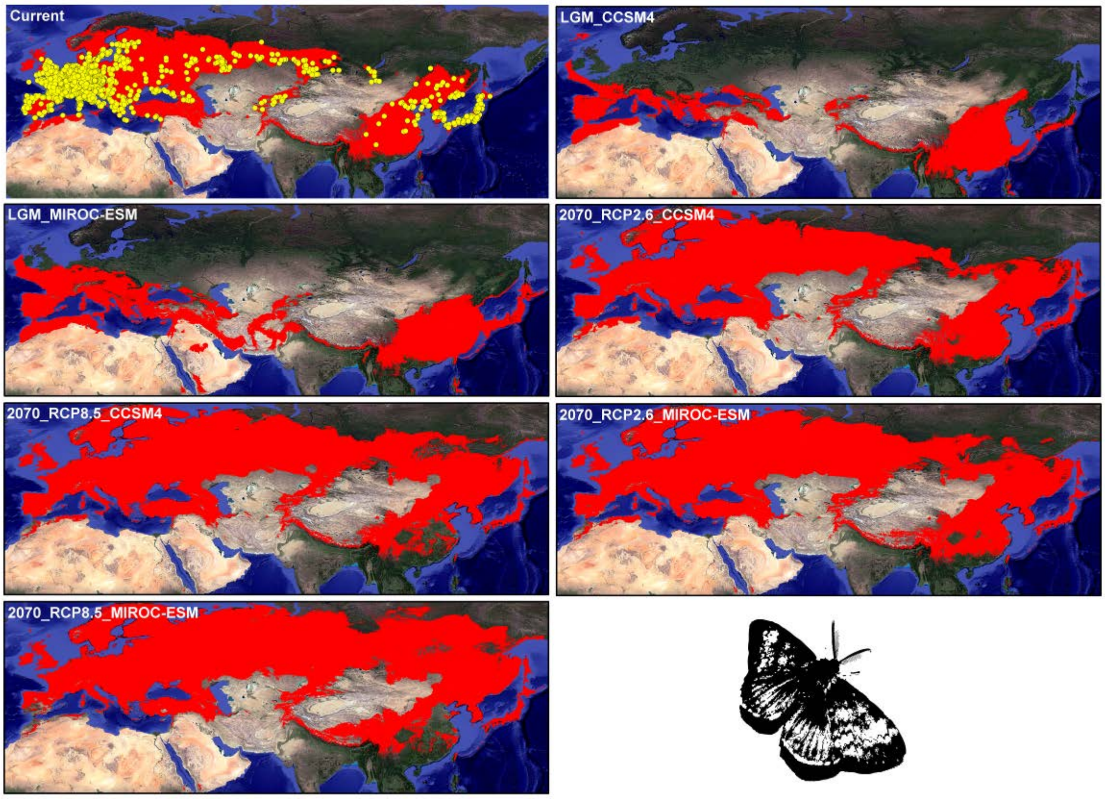

Supplementary fig. S20 Modelled suitable areas of *Meles meles* throughout the Eurasia under the 10 percentile training presence threshold from the current climatic condition, the LGM and the two greenhouse gas emission scenarios (RCP 2.6 and RCP 8.5) for the year 2070 under the CCSM4 and MIROC-ESM. Red colours indicate habitat suitability areas. Occurrence localities (yellow dots) are used for ecological niche modelling.

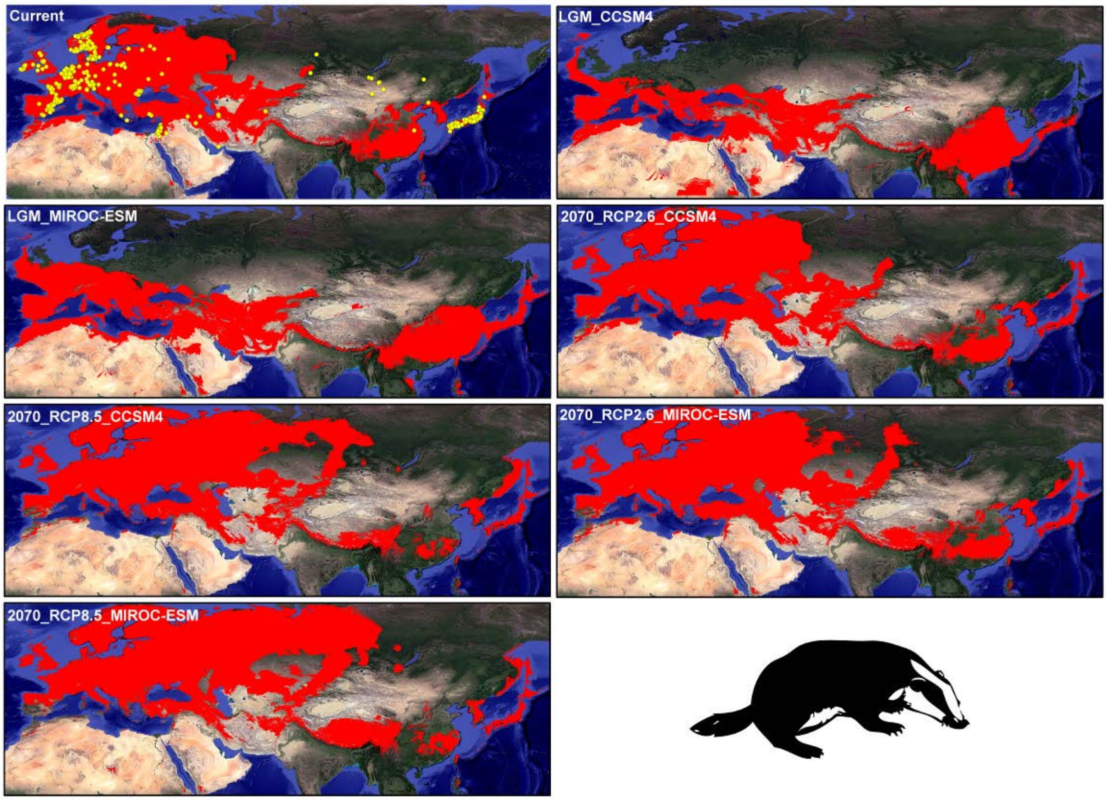

Supplementary fig. S21 Modelled suitable areas of *Pica pica* throughout the Eurasia under the 10 percentile training presence threshold from the current climatic condition, the LGM and the two greenhouse gas emission scenarios (RCP 2.6 and RCP 8.5) for the year 2070 under the CCSM4 and MIROC-ESM. Red colours indicate habitat suitability areas. Occurrence localities (yellow dots) are used for ecological niche modelling.

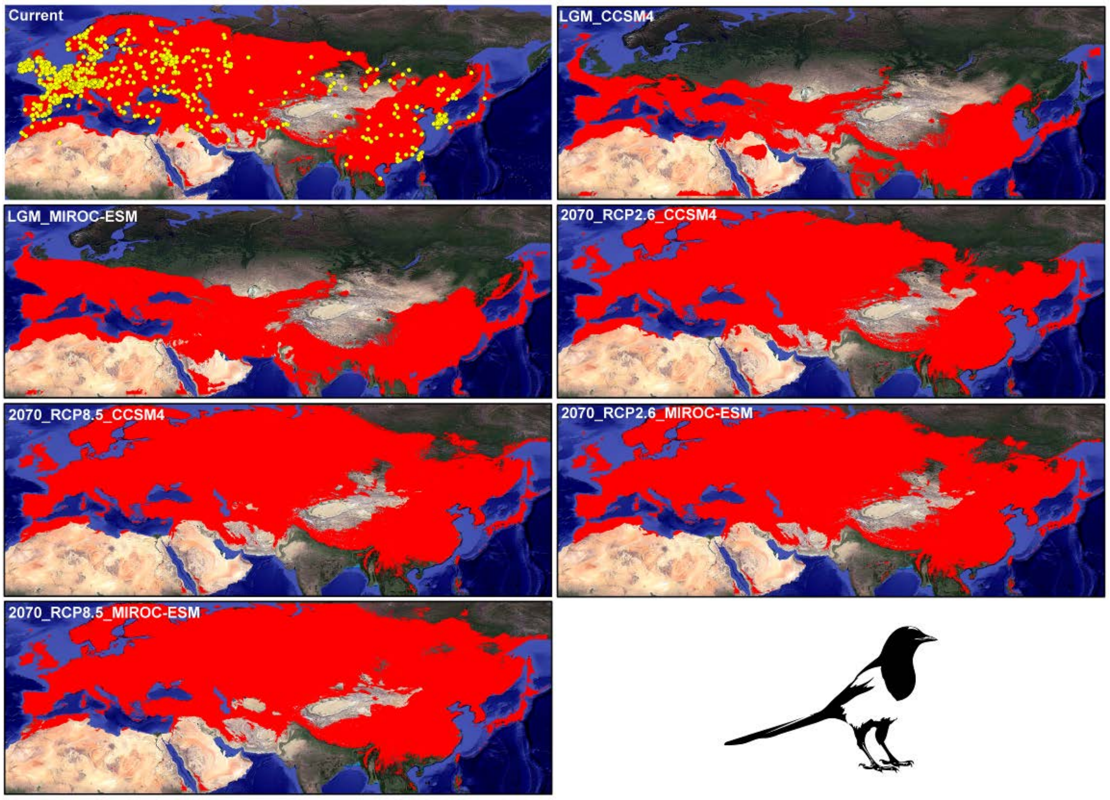

Supplementary tables

Supplementary table S5 Nucleotide polymorphisms in each geographical population of *A. paludum* based on the 32,114 SNPs of the ddRAD_95 SNPs dataset. H_O, observed heterozygosity; H_E, expected heterozygosity; π_S, nucleotide diversity.

Sample	Lat.	Long.	ddRAD_95 SNPs dataset			
			Sample size	H_O	H_E	π_S
**Western lineage**						
AZER	38.45	48.76	8	0.30541	0.31435	0.030553
BG	43.02	27.82	4	0.38764	0.40570	0.038825
BK	45.31	16.84	5	0.35000	0.36637	0.029876
DKZE	55.46	11.72	25	0.29072	0.29731	0.031619
ELSR	47.42	31.87	3	0.53333	0.47644	0.033999
FRAN	48.86	2.35	13	0.22711	0.31227	0.035209
GEOR	42.68	41.51	10	0.30333	0.32865	0.030029
ITAJ	46.13	12.24	15	0.26246	0.27249	0.031506
SWRA	46.73	15.82	5	0.36939	0.38186	0.035879
LMNS	45.73	24.22	17	0.23760	0.26016	0.035860
UZBK	41.53	69.69	16	0.36779	0.34468	0.012013
XJAL	48.07	86.33	11	0.25331	0.27849	0.036916
NMBT	40.71	110.68	10	0.22275	0.24075	0.041837
**Eastern Lineage**						
HLQQ	47.34	123.93	10	0.18268	0.21025	0.053431
SXWN	34.49	109.83	9	0.17396	0.19960	0.046531
SCMY	31.69	104.28	8	0.22005	0.25691	0.070214
HNBS	19.09	109.09	10	0.19880	0.22855	0.043226
LBLB	19.76	101.99	10	0.36040	0.34883	0.038631
THAN	19.09	97.93	10	0.22908	0.26405	0.039693
TW	22.89	120.55	10	0.18889	0.23099	0.044469
GZQN	26.25	107.31	10	0.19053	0.21053	0.047059
FJXM	24.76	118.13	10	0.19358	0.22102	0.047782
YNTC	25.12	98.56	10	0.18714	0.23226	0.036866
ZJZS	30.08	122.06	10	0.22460	0.25752	0.038813

Supplementary table S6 Hierarchical analyses of molecular variance (AMOVA) for *A. paludum* grouped into two genetic clusters (Western and Eastern lineages).

Source of variation	Percentage of variation	Fixation indice	*p* value
**Nuclear SNPs data**			
Among groups	26.03	F_{CT}=0.26033	<0.01*
Among populations within groups	10.52	F_{SC}=0.14221	<0.01*
Within populations	63.45	F_{ST}=0.36551	<0.01*
**Mitochondrial data**			
**COI+COII**			
Among groups	37.25	F_{CT}=0.37255	<0.01*
Among populations within groups	9.83	F_{SC}= 0.15673	<0.01*
Within populations	52.91	F_{ST}= 0.47089	<0.01*
**Mitochondrial genome (13 PCGs)**			
Among groups	36.44	F_{CT}=0.36437	<0.01*
Among populations within groups	13.64	F_{SC}= 0.21460	<0.01*
Within populations	49.92	F_{ST}= 0.50077	<0.01*

Supplementary table S7 Results from demographic model selection analyses in fastsimcoal2 for the two lineages (W and E) of *A. paludum*. Model numbers correspond to those in Supplementary fig. S1. Model best supported by the data are highlighted in bold. *n*, number of parameters.

**Model**	**Description**	***n***	**DeltaL**	**AIC**	**△ AIC**	**AIC weight**
M1	Isolation model	4	123.76	5024.99	349.36	1.372271e-76
M2	Isolation with Migration (IM) model with symmetric migration	6	66.69	4768.21	92.58	7.879677e-21
M3	IM model with migration from West to East	5	78.68	4823.41	147.78	8.127945e-33
M4	IM model with migration from East to West	5	83.32	4842.78	167.15	5.056368e-37
M5	IM model with symmetric migration, followed by recent isolation	7	129.61	5059.95	384.32	3.515381e-84
M6	IM model with isolation, followed by secondary contact and symmetric migration	7	46.16	4675.63	0	1
M7	IM model with migration from West to East, followed by recent isolation	6	129.58	5059.82	384.19	3.75147e-84
M8	IM model with isolation, followed by secondary contact and migration from West to East	6	73.75	4802.69	127.06	2.566088e-28
M9	IM model with migration from East to West, followed by recent isolation	6	130.54	5062.23	386.60	1.124286e-84
M10	IM model with isolation, followed by secondary contact and migration from East to West	6	69.50	4781.14	105.51	1.226859e-23

Supplementary table S8 Parameter estimates for the best-fit demographic model (Model 6). All estimates assume diploid cells, a 0.5-year generation time, and a nuclear mutation rate of 3.5E-9 per site per generation. Point estimates are provided with 95% confidence intervals in parentheses. Point estimates are those identified in the best-fit run of the 50 model selection replicates. Abbreviations include: N_e = effective population size.

Parameter	Description	Estimate
Effective population sizes		
W_N_e	West N_e	7,024,866 (6,285,572 – 7,764,159)
E_N_e	East N_e	549,728 (491,442 – 608,014)
N_0	Ancestral N_e of West+East	5,666 (4,940 – 6,393)
Times estimates		
T_0	Divergence time between West and East	383,708 (343,278 – 424,139)
T_1	Contact time between West and East	2,870 (2,529 – 3,212)
Migration probabilities		
M_{EW}	Migration rates from East into West	1.2E-6 (1.1E-6 – 1.3E-6)
M_{WE}	Migration rates from West into East	3.6E-7 (3.4E-7 – 3.8E-7)

Supplementary table S9 Principal components analysis (PCA) of five environmental variables associated with occurrence of *A. paludum* including the two lineages and the hybrid populations (i.e., NMBT and HLQQ). Eigenvalues for the most important variables (> 0.8) in PCA are in bold.

		**Factor loadings**		
**Variables**	**Description**	PC-1	PC-2	PC-3
**BIO1**	Annual mean temperature	0.705	0.542	-0.273
**BIO8**	Mean temperature of wettest quarter	**0.820**	-0.049	0.558
**BIO12**	Annual precipitation	0.671	0.647	-0.075
**BIO14**	Precipitation of driest month	-0.501	**0.818**	0.204
**BIO15**	Precipitation seasonality	**0.846**	-0.433	-0.132
**Eigenvalue**		2.586	1.572	0.451
**Percentage variance**		51.730	31.446	9.023
**Cumulative percentage variance**		51.730	83.176	92.199

Supplementary methods

Mitochondrial DNA sequencing

A total of 390 out of 409 individuals were sequenced for COI and COII fragments (supplementary table S1, Supplementary Material online). PCR-specific primers and PCR procedures followed our previous work (Ye et al. 2014; Ye et al. 2018). PCR products were sent to BGI (Beijing Genomics Institute) for Sanger sequencing in both directions. Acquired sequences were aligned with ClustalW (Larkin et al. 2007) for multiple alignments under default settings and visually proofread in Bioedit v7.0 (Hall 1999). Furthermore, 112 individuals representing 22 populations were selected to generate the whole mitochondrial genome (supplementary table S2, Supplementary Material online). The whole mitochondrial genome was obtained using the Illumina HiSeq 2000 platform with a 250-bp insert size and a paired-end 150-bp sequencing strategy at Novogene. 2 Gb of clean data for each individual was obtained. The individual read files were then mapped in Geneious Prime 2020.0.5 (https://www.geneious.com/) against the 13 mitochondrial protein-coding genes (PCGs) of the published mitogenome of *A. paludum* (GenBank accession no.: NC_012841). For the mapping parameters, medium–low sensitivity was selected because it is suitable for large numbers of high-throughput sequencing reads. The consensus threshold was set to the highest quality, as recommended by the software. Reads that mapped to multiple locations were ignored. Consensus mapping required a minimum depth of three reads, and the consensus sequences of each individual were trimmed to the length of the reference mitochondrial PCGs. Finally, we aligned each PCG-mapped matrix for the 112 individuals with muscle 3.7 (Edgar 2004) under the default settings and concatenated the 13 aligned PCG matrices.

ddRAD-seq Library preparation, sequencing and variant calling

A double-digest restriction site-associated DNA sequencing (ddRAD-seq) library was prepared to obtain nuclear SNPs following Peterson's protocol (Peterson et al. 2012). Briefly, 360 ng DNA was double-digested using *EcoRI* and *MspI* restriction enzymes (New England Biolabs, Ipswich, MA, USA). We used five-base barcode adapters to uniquely mark each individual, each of which differed by at least three nucleotides. The ligation products were pooled and run through a Pippin Prep (Sage Science, USA) to select fragments between 250 and 600 bp, followed by the amplification of the size-selected pools with 10–12 cycles using PCR with Illumina indexed primers. Amplified DNA fragments from each sample were purified using AMPure XP magnetic beads (Beckman Coulter Inc, Indianapolis, IN, USA) and quantified with an Agilent 2100 bioanalyzer (Agilent Technologies, Santa

Clara, CA, USA). Finally, 150 bp paired-end reads were generated on an Illumina HiSeqX10 platform at the Novogene Sequencing Center & CAP and ISO Lab, Tianjin, China. The raw data were filtered, and the sequences of the adapter and low-quality data were removed, resulting in a clean dataset for the subsequent analysis. We processed ddRAD-seq clean reads using the ipyrad 0.9.42 pipeline (Eaton and Overcast 2017). The reads with an average Phred score offset of 33 were retained, and a clustering threshold of 85% was used. We set a minimum read depth of 6 for calling consensus sequences within samples, and the maximum number of polymorphic sites in a final locus was set to 20. All other parameters were left at the default values. A ddRAD_95 SNPs dataset was generated, and each locus was required to be present in at least 95% of individuals (i.e., 5% missing samples per locus). To avoid linkages across sites within the same locus, one random SNP was sampled from each locus, and the ddRAD_95 USNPs dataset was finally generated for the downstream analyses.

Whole-genome sequencing and variant calling

To further investigate the historical demographic changes and scanning signatures of selection between the western and eastern lineages of *A. paludum*, we chose one individual from each population and shotgun sequenced the genomes of 21 individuals (6.51± 0.89 Gb, range 5.62–7.4 Gb) (supplementary table S4, Supplementary Material online). Of these, two individuals (AZER1 and ZJZS9) from the western and eastern lineages, respectively, were shotgun sequenced at 24.53 GB and 22.46 GB for pairwise sequential Markovian coalescent (PSMC) analyses. DNA libraries with 350 bp insertions were constructed. All libraries were sequenced using Illumina HiSeqX10 with a paired-end read length of 150 bp in Novogene Sequencing Center & CAP and ISO Lab, Tianjin, China. The raw reads were processed to remove adapter sequences and low-quality data. Because a reference genome is currently not available for *A. paludum*, quality-controlled clean reads of all individuals were mapped to a reference genome of a closely related species from the genus *Gerris* (Damgaard and Cognato 2006; Ye et al. 2018; *Gerris buenoi*, Armisén et al. 2018, GenBank accession no.: GCA_001010745.2) using BWA 0.7.17 (Li and Durbin 2009). SNPs calling was performed using GATK v4.1.9.0 (McKenna et al. 2010). PCR duplicates were removed before variant calling using SAMtools 1.12 (Li et al. 2009) with the ‘rmdup’ command. We filtered SNPs named WGS_SNPs dataset using VCFtools 0.1.15 (Danecek et al. 2011) using the following criteria: (i) quality value≥30; (ii) retain only biallelic SNPs; (iii) genotype depth≥5; (iv) no missing genotypes; (v) filter out SNPs differing in the reference species but otherwise homogeneous.

Mantel test

Isolation by distance (IBD), isolation by environment (IBE) and isolation by resistance-climate (IBR) tests were investigated based on the ddRAD_95 SNPs dataset. These tests were performed by examining the correlation between genetic distance and geographical/environmental/resistance-climatic distances using the Mantel test, as implemented in the R package Adegenet (Jombart 2008), with 10,000 permutations. Genetic distance was estimated as $F_{ST}/(1 - F_{ST})$, in which values of pairwise F_{ST} between populations were calculated using Arlequin 3.5 (Excoffier and Lischer 2010). Pairwise geographic distances among populations were calculated using the *pointDistance* function in the R package raster (Hijmans 2020). IBE was represented as climatic dissimilarity, estimated using five climatic variables (i.e., BIO1, BIO8, BIO12, BIO14 and BIO15) applied in the ENM analysis (see ENM analysis). We extracted the climate variables based on sample site coordinates using the R package raster (Hijmans 2020). To reduce collinearity, a principal component analysis (PCA) was performed on the extracted climate variables among populations using the R package ade4 (Dray and Dufour 2007). The first two principal components (PC1 and PC2) were used to calculate Euclidean environmental distances among locations. For the IBR analysis, we first applied the least cost distances (LCDs) method to visualize dispersal corridors of *A. paludum* across the landscapes (habitat heterogeneity) for the two periods (i.e., the current and the future condition). The future condition was selected under RCP 8.5 (the maximum greenhouse gas emission scenario) for the year 2070 under the CCSM4 model. We created a resistance layer by inverting the climate suitability surfaces estimated by the ENM analysis (see ENM analysis), which acted as a cost distance raster. LCDs for the landscape resistance surfaces were calculated among all pairwise population combinations using the *costDistance* function in the R package gdistance (van Etten 2017). Under this framework, grid values range from 0–1, with 1 corresponding to no landscape resistance.

Historical demographic changes

We used three datasets to reconstruct the recent demographic trajectories for the western and eastern lineages of *A. paludum*. The first dataset included mitochondrial COI gene fragments from 390 individuals, the second dataset included ddRAD_95 SNPs from 249 individuals, and the third dataset included assembled whole genome sequencing contig files from 2 individuals (AZER1 and ZJZS9) with a high sequencing depth (~25-fold). For the first dataset, the Bayesian skyline plot (BSP) analysis generated in BEAST 2.6.3 (Bouckaert et al. 2014) was reconstructed. We employed an accepted

substitution rate of 0.004–0.008 site/million years for the COI gene in semiaquatic Heteroptera (Damgaard and Zettel 2003; Ye et al. 2014) and a generation time of 0.5 years (Andersen 1982). The program ModelFinder (Kalyaanamoorthy et al. 2017) was used to infer the best models of substitution via the BIC criterion. HKY+F+G4 was selected as the best-fitting model for the western lineage, while TN+F+G4 was chosen for the eastern lineage. We assigned a lognormal relaxed clock with uncorrelated rates to the clock model. The final MCMC chains were run for 100 million generations with sampling every 1000 generations. The ESS values (>200) were checked, and the demographic changes over time were reconstructed using Tracer 1.7 (Rambaut et al. 2018). For the second dataset, we used the stairway plot method (Liu and Fu 2015) to investigate the detailed population demographic history for each lineage utilizing a one-dimensional unfolded SFS. We generated a SFS for each genetic lineage (west and east) using easySFS. For the third dataset, we used the PSMC model (Li and Durbin 2011) to reconstruct the recent demographic trajectories based on individual contig files of the genomic data for each lineage (AZER1 and ZJZS9). MEGAHIT 1.2.8 (Li et al. 2015) was used for de novo assembly using the default settings. Since no reference genome for species of *Aquarius* was available in GenBank, we used the contig files that were obtained in the above assemblies from MEGAHIT to regenerate two pseudochromosomes for the two individuals. We then mapped the clean reads back to the newly generated pseudochromosome data individually. The whole-genome diploid consensus sequences for each individual were generated by SAMtools 1.12 (Li et al. 2009) and bcftools 1.7 (Li et al. 2009) using the parameter C50. Sites with sequencing depths<10 and>100 (vcfutils.pl vcf2fq -d 10 -D 100) were removed to reduce the probability of false-positives. The PSMC parameter (psmc -N25 -t15 -r5 -p “4+25×2+4+6”) was used to infer the historical effective population size. To determine the variance in *Ne* estimates, we performed 100 bootstraps for each sample. The assumed nuclear mutation rate of 3.5E-9 per site per generation and a generation time of 0.5 years were used for the second and third datasets, which was consistent with those used in the demographic model testing.

References

Andersen NM. 1982. The semiaquatic bugs (Hemiptera, Gerromorpha): phylogeny, adaptations, biogeography and classification. Entomonograph 3. Klampenborg: Scandinavian Science Press Ltd.

Armisén D, Rajakumar R, Friedrich M, Benoit JB, Robertson HM, Panfilio KA, Ahn SJ, Poelchau MF, Chao H, Dinh H, et al. 2018. The genome of the water strider *Gerris buenoi* reveals expansions of gene repertoires associated with adaptations to life on the water. *BMC Genomics* 19:832.

Bouckaert R, Heled J, Kühnert D, Vaughan T, Wu CH, Xie D, Suchard MA, Rambaut A, Drummond AJ. 2014. BEAST 2: a software platform for Bayesian evolutionary analysis. *PLoS Comput. Biol.* 10: e1003537.

Damgaard J, Cognato AI. 2006. Phylogeny and reclassification of species groups in *Aquarius* Schellenberg, *Limnoporus* Stål and *Gerris* Fabricius (Insecta: Hemiptera-Heteroptera, Gerridae). *Syst Entomol*. 31(1):93–112.

Damgaard J, Zettel H. 2003. Genetic diversity, species phylogeny and historical biogeography of the *Aquarius paludum* group (Heteroptera: Gerridae). *Insect Syst Evol*. 34(3):313–328.

Danecek P, Auton A, Abecasis G, Albers CA, Banks E, DePristo MA, Handsaker RE, Lunter G, Marth GT, Sherry ST, et al. 2011. 1000 Genomes Project Analysis Group. The variant call format and VCFtools. *Bioinformatics*. 27(15):2156–2158.

Dray S, Dufour AB. 2007. The ade4 package: Implementing the duality diagram for ecologists. *J Stat Softw*. 22(4):1–20.

Eaton DA, Overcast I. 2020. Ipyrad: interactive assembly and analysis of RADseq. Retrieved from http://ipyrad.readthedocs.io/.

Edgar RC. 2004. MUSCLE: a multiple sequence alignment method with reduced time and space complexity. *BMC Bioinformatics* 5(1):1–19.

Excoffier L, Lischer HE. 2010. Arlequin suite ver 3.5: a new series of programs to perform population genetics analyses under Linux and Windows. *Mol Ecol*. 10(3):564–567.

Fields PD, Obbard DJ, McTaggart SJ, Galimov Y, Little TJ, Ebert D. 2018. Mitogenome phylogeographic analysis of a planktonic crustacean. *Mol Phylogenet Evol*. 129:138–148.

Hall T. 1999. BioEdit: a user-friendly biological sequence alignment editor and analysis program for Windows 95/98/NT. *Nucleic Acids Symp Ser*. 45:95–98.

Hijmans RJ. 2020. raster: Geographic analysis and modeling. R package version 3.0-13. Retrieved from http://CRAN.R-proje ct.org/package=raster.

Jombart T. 2008. adegenet: a R package for the multivariate analysis of genetic markers. *Bioinformatics* 24(11):1403–1405.

Kalyaanamoorthy S, Minh BQ, Wong TK, Von Haeseler A, Jermiin LS. 2017. ModelFinder: fast model selection for accurate phylogenetic estimates. *Nat Methods*. 14(6):587–589.

Krehenwinkel H, Graze M, Rödder D, Tanaka K, Baba YG, Muster C, Uhl G. 2016. A

phylogeographical survey of a highly dispersive spider reveals eastern Asia as a major glacial refugium for Palaearctic fauna. *J Biogeogr*. 43(8):1583–1594.

Li D, Liu CM, Luo R, Sadakane K, Lam TW. 2015. MEGAHIT: an ultra-fast single-node solution for large and complex metagenomics assembly via succinct de Bruijn graph. *Bioinformatics* 31(10):1674–1676.

Li H, Durbin R. 2009. Fast and accurate short read alignment with Burrows-Wheeler transform. *Bioinformatics* 25(14):1754–1760.

Li H, Durbin R. 2011. Durbin, Inference of human population history from individual whole-genome sequences. *Nature* 475:493–496.

Li H, Handsaker B, Wysoker A, Fennell T, Ruan J, Homer N, Marth G, Abecasis G, Durbin R. 2009. The sequence alignment/map format and SAMtools. *Bioinformatics* 25(16): 2078–2079.

Liu X, Fu YX. 2015. Exploring population size changes using SNP frequency spectra. *Nat Genet*. 47(5): 555–559.

Marmi J, Lopez‐Giraldez F, Macdonald DW, Calafell F, Zholnerovskaya E, Domingo‐Roura X. 2006. Mitochondrial DNA reveals a strong phylogeographic structure in the badger across Eurasia. *Mol Ecol*. 15(4):1007–1020.

McKenna A, Hanna M, Banks E, Sivachenko A, Cibulskis K, Kernytsky A, Garimella K, Altshuler D, Gabriel S, Daly M, et al. 2010. The Genome Analysis Toolkit: a MapReduce framework for analyzing next-generation DNA sequencing data. *Genome Res*. 20(9):1297–1303.

Rambaut A, Drummond AJ, Xie D, Baele G, Suchard MA. 2018. Posterior summarization in Bayesian phylogenetics using Tracer 1.7. *Syst Biol*. 67(5):901–904.

Song G, Zhang RY, Alström P, Irestedt M, Cai TL, Qu YH, Ericson PGP, Fjeldså J, Lei FM. 2018. Complete taxon sampling of the avian genus *Pica* (magpies) reveals ancient relictual populations and synchronous Late‐Pleistocene demographic expansion across the Northern Hemisphere. *J Avian Biol*. 49(2):jav-01612.

van Etten J. 2017. R package gdistance: Distances and routes on geographical grids. *J Stat Softw*. 76(13):1–21.

Wu YK, Molongoski JJ, Winograd DF, Bogdanowicz SM, Louyakis AS, Lance DR, Mastro VC, Harrison RG. 2015. Genetic structure, admixture and invasion success in a Holarctic defoliator, the gypsy moth (*Lymantria dispar*, Lepidoptera: Erebidae). *Mol Ecol*. 24(6):1275–1291.

Ye Z, Yuan JJ, Li M, Damgaard J, Chen PP, Zheng CG, Yu HB, Fu SY, Bu WJ. 2018. Geological effects influence population genetic connectivity more than Pleistocene glaciations in the water strider *Metrocoris sichuanensis* (Insecta: Hemiptera: Gerridae). *J Biogeogr*. 45(3):690–701.

Ye Z, Zhen YH, Damgaard J, Chen PP, Zhu L, Zheng CG, Bu WJ. 2018. Biogeography and diversification of Holarctic water striders: Cenozoic temperature variation, habitat shifting and multiple intercontinental dispersals. *Syst Entomol*. 43(1):19–30.

Ye Z, Zhu GP, Chen PP, Zhang DL, Bu WJ. 2014. Molecular data and ecological niche modelling reveal the Pleistocene history of a semi–aquatic bug (*Microvelia douglasi douglasi*) in East Asia. *Mol Ecol*. 23(12):3080–3096.
